# Creating diversity in mammalian facial morphology: a review of potential developmental mechanisms

**DOI:** 10.1186/s13227-018-0103-4

**Published:** 2018-06-14

**Authors:** Kaoru Usui, Masayoshi Tokita

**Affiliations:** Department of Biology, Faculty of Science, Toho University, 2-2-1 Miyama, Funabashi, Chiba 274-8510 Japan

**Keywords:** Mammals, Craniofacial morphology, Diversity, Transgenic mice, Bats, Facial processes, Neural crest, Ectomesenchyme, Bone, Orofacial cleft

## Abstract

Mammals (class Mammalia) have evolved diverse craniofacial morphology to adapt to a wide range of ecological niches. However, the genetic and developmental mechanisms underlying the diversification of mammalian craniofacial morphology remain largely unknown. In this paper, we focus on the facial length and orofacial clefts of mammals and deduce potential mechanisms that produced diversity in mammalian facial morphology. Small-scale changes in facial morphology from the common ancestor, such as slight changes in facial length and the evolution of the midline cleft in some lineages of bats, could be attributed to heterochrony in facial bone ossification. In contrast, large-scale changes of facial morphology from the common ancestor, such as a truncated, widened face as well as the evolution of the bilateral cleft possessed by some bat species, could be brought about by changes in growth and patterning of the facial primordium (the facial processes) at the early stages of embryogenesis.

## Morphological diversity in mammalian faces

**Fig. 1:**
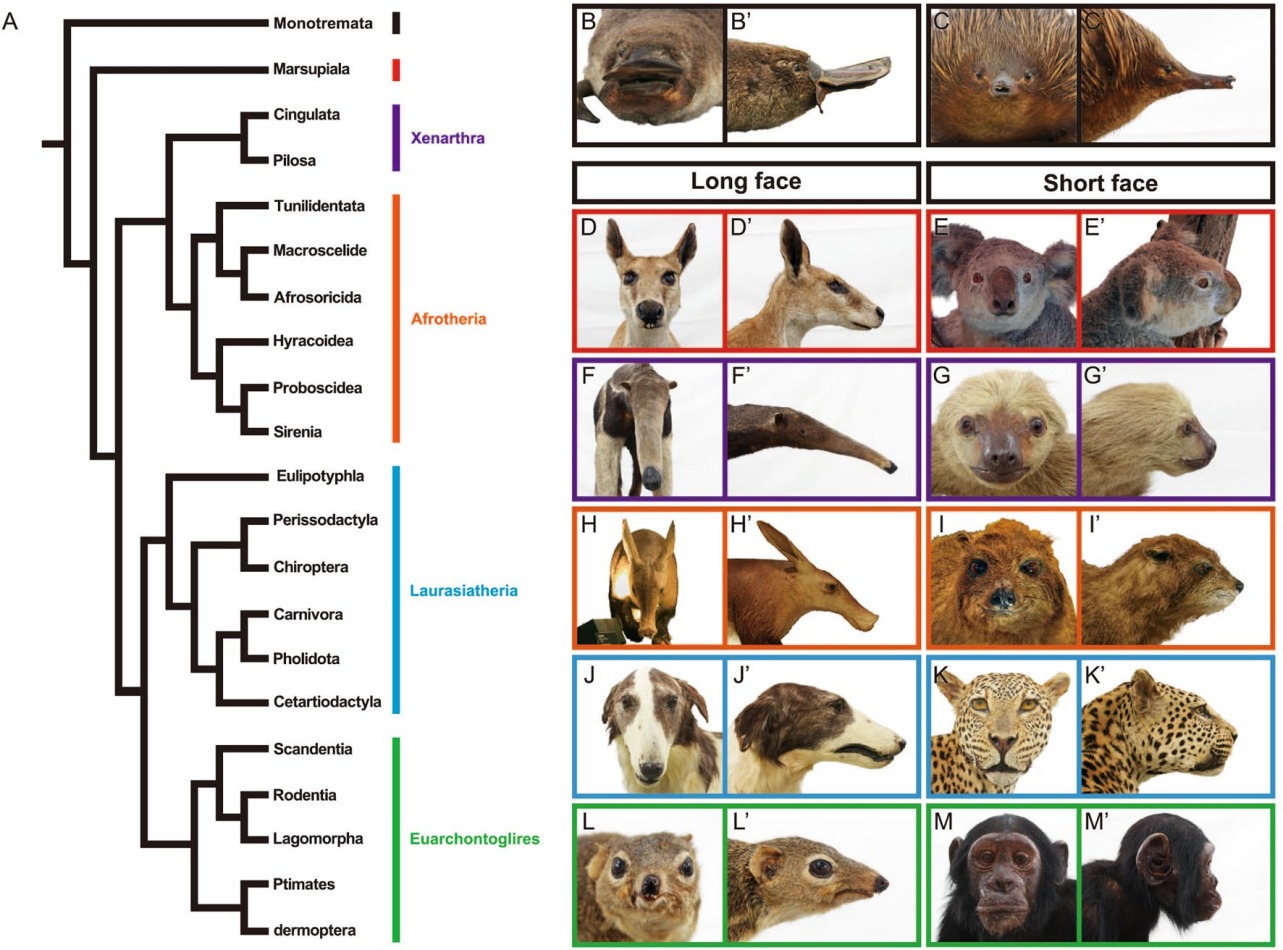
Phylogeny of mammals and diversity of their craniofacial morphology. *A*, phylogeny of living mammals adapted from Tarver et al. [11], with phylogeny of Laurasiatheria based on Chen et al. [101]. *B–M′*, frontal and lateral views of mammalian heads: *B* and *B′*, platypus (*Ornithorhynchus anatinus*); *C* and *C′*, echidna (*Tachyglossus aculeatus*); *D* and *D′*, red kangaroo (*Macropus rufus*); *E* and *E′*, koala (*Phascolarctos cinereus*); *F* and *F′*, giant anteater (*Myrmecophaga tridactyla*); *G* and *G′*, Linnaeus’s two-toed sloth (*Choloepus didactylus*); *H* and *H′*, aardvark (*Orycteropus afer*); *I* and *I′*, rock hyrax (*Procavia capensis*); *J* and *J′*, domestic dog (Borzoi) (*Canis lupus familiaris*); *K* and *K′*, leopard (*Panthera pardus*); *L* and *L′*, common tree shrew (*Tupaia glis*); *M* and *M′*, common chimpanzee *(Pan troglodytes*)

Mammals (class Mammalia) are one of the major groups of vertebrates, containing over 5400 living species as well as abundant extinct species [1–4]. Living mammals consist of three major clades: monotremes (order Monotremata), marsupials (infraclass Marsupialia), and placentals (infraclass Placentalia; Fig. 1). Recent phylogenetics, including comparative phylogenomic studies, have lead to a general consensus concerning the deeper branches of the mammalian evolutionary tree, for example identifying four major clades within placentals: Xenarthra, Afrotheria, Laurasiatheria, and Euarchontoglires [5–11].

Mammals have evolved diverse morphologies to adapt to a wide range of ecological niches [3, 4]. The morphological diversity of mammalian heads is especially remarkable, possibly due to the head’s fundamental role in sensing, communication, and feeding [12–18] (Fig. 1). For example, both long- and short-faced taxa are recognized in each mammalian group (Fig. 1). Craniofacial morphology in mammals has been quantitatively evaluated in each group by comparative morphological analyses, including modern geometric morphometrics (summarized in Table 1).

**Table 1:** Diversity of craniofacial morphology in mammals and recent studies evaluating this diversity using landmark-based geometric morphometrics

Clade	Remarks on diversity of craniofacial morphology	Landmark-based geometric morphometric studies
Monotremata	All extant monotremes have a toothless bill covered by electro- and mechano-receptors. The platypus has a flat, widened, duck-like bill. Echidna bills are more pointed, slender compared to platypus bills	None
Marsupialia	The viscerocranium, which includes the early-ossifying bones of the oral region, is morphologically less diverse than in placentals. The level of disparity of late-ossifying neurocranium is equivalent with that in placentals. This suggests that the ossification of marsupial oral bones is more constrained compared to placentals	[102–105]
*Xenarthra*		
Cingulata	Armadillo skulls are elongated anteroposteriorly and flattened dorsoventrally. The zygomatic arch is complete, differing from those of another xenarthran lineage, Pilosa. The dentary bone is thin and long. Variation in skull shape is only described in the family Pampatheriidae which is an extinct group of Cingulata. Skull shape is highly conserved among extant members	[106]
Pilosa	The suborder Forivora (sloths), which consists of Bradypodidae (three-toed sloth) and Megalonychidae (two-toed sloths), has a short, high skull with a strongly reduced rostrum. The zygomatic arch is robust but incomplete. The skulls of three-toed and two-toed sloths are distinct to one another according to morphometric analyses. Three-toed sloth skulls have a relatively shortened rostrum and no diastema. The suborder Vermilingua (anteaters) has a specialized skull for eating small insects; the skull is highly elongated and has no tooth. Its pointed rostrum encases a long tongue. The zygomatic arch is incomplete	[107, 108]
*Afrotheria*		
Tubulidentata	Aardvark skulls are elongated anteroposteriorly, accompanied by long and slender dentary bones. The nasal bone is triangular in shape. The frontal bones expand dorsally in front of the orbit as a result of a highly developed nasal chamber. The zygomatic arch is complete but slender. There is no postorbital bar	None
Macroscelidea	Macroscelidea species have a tall, dome-shaped cranium. The zygomatic arch is complete. The rostrum is long. Macroscelidae consists of two subfamilies: Rhynchocyoninae and Macroscelidinae. Rhynchocyoninae species have a relatively large skull with nasal bones having partially ossified tips. The bony palate is not perforated. Macroscelidinae species have a relatively smaller skull and wholly cartilaginous nasal bone tips. The bony palate has some holes	[109, 110]
Afrosoricida	Afrosoricida consists of two families: Tenrecidae (tenrecs) and Chrysochloridae (golden moles). Tenrec skulls have a long, slender rostrum. The jugal bone is absent and the orbital bone is usually small. The skull of golden moles is abruptly conical, its anterior portion is pointed, and its posterior portion widened. The zygomatic arch is formed by an elongated process of the maxilla, and the occipital area contains the tabular bones, which are not typical in mammals. Tenrec skulls are less morphologically diverse than those of golden moles. It is suggested that the similarities in skull morphology among the speciose genus *Microgale* masks morphological diversity among the rest of the family	[111]
Hyracoidea	All four extant hyrax species have short skulls and deep dentary bones. The skull has a postorbital bar, which is sometime complete (*Dendrohyrax*) and sometime incomplete (*Heterohyrax* and *Procavia*)	None
Proboscidea	All extant elephant species *(Loxodonta* and *Elephas*) have short, tall skulls which are pneumatized particularly in the cranial roof, thereby reducing cranium weight. Skulls bears two tusks derived from the second incisors of the upper jaw	[112]
Sirenia	The skulls of Sirenia species are highly specialized for aquatic life, including adaptations such as deep dentary bones. Sirenia consists of two families: Dugongidae and Trichechidae. In Dugongidae skulls, the premaxilla bones are relatively larger, the nasal bones are absent, and the nasal cavity is shortened. In Trichechidae skulls, the premaxilla bones are small, the nasal bones are present, and the nasal cavity is elongated. Within Trichechidae, *Trichechus inunguis* is distinct in skull shape. The skull shape of *T. senegalensis*, *T. manatus manatus*, and *T. m. latirostris* are more similar to each other. Within *T. manatus*, geographic variations in skull morphology, perhaps caused by geographic isolation, are reported	[113]
*Laurasiatheria*		
Eulipotyphla	Disparity in skull morphology among eulipotyphylans may be explained by phylogeny rather than ecology. In the genus *Sorex*, similarities and differences in skull shape between species are proportional to the phylogenic distance between them. Similarly, the degree of morphological variation in the dentary bone between talpid species corresponds to the phylogenetic distance between the species	[114–119]
Perissodactyla	Perissodactyla skulls are adapted to an herbivorous diet. Extant Perissodactyla consists of three families: Equidae, Tapiridae, and Rhinocerotidae, and all have a long skull with an elongated face and large cheek teeth adapted for grinding coarse vegetation. Equid skulls are generally flat in a mediolateral direction, with long, deep rostrums. The skulls of the Tapiridae have a well-developed sagittal crest, rostrally positioned orbital bones, and a small cranium with a reduced posterior region. Rhinocerotidae have a thickened, enlarged nasal bone which extends anteriorly beyond the anterior margin of the premaxilla bone. The occipital bone is unusually high where the neck muscles attach to sustain the heavy head	None
Chiroptera	Bat skulls are morphologically highly diverse. However, the degree of morphological disparity in skull shape is not the same among taxa. The family Pteropodidae, which lost the ability to echolocate, have large orbits accompanied with a well-developed postorbital bar. The rostrum is morphologically uniform despite variation in diet between species. The family Phyllostomidae shows a high level of variation in skull morphology explained by a diversity of diets. Nectarivorous species possess an elongated face while fruigivorous species have a shortened face. Skull morphology of the family Vespertilionidae is highly conserved, although it is the most speciose group in the order	[82, 85, 86, 94, 120, 121]
Carnivora	Carnivoran skulls are characterized by an expanded braincase in which the frontal-parietal suture is located posteriorly relative to the postorbital constriction, as well as fully or partially ossified ectotympanic bones that are firmly fused to the skull. Carnivoran skulls are highly varied corresponding to different diets. In general, felid species have a shorter rostrum for production of higher bite force, while canid species typically have a longer rostrum with a large nasal chamber associated with a well-developed olfactory sense. The pinnipeds, semiaquatic marine mammals, usually have a short rostrum, and enlarged orbits	[53, 122–146]
Pholidota	All extant pangolin species have a long, narrow, toothless skull. The dentary bone is narrow and slender as well. The surface of the cranium is smooth without any ridges or crests. The zygomatic arch is present but incomplete. The postorbital bar is absent	None
Cetartiodactyla	The skulls of Cetartiodactyla usually have a long rostral portion. The postorbital bar is always present. When horns are present, they are most often formed on the frontal bones. The extant Cetartiodactyla consists of the suborder Suina (pigs and peccaries), the infraorder Cetacea (whales), the infraorder Ancodonta (hippos), and the suborder Ruminantia (cows, goats giraffes, deers etc.). Suiforme skulls are distinct from those of other cetartiodactyls, having a posteriorly extended squamosal bone that contacts the exoccipital bone. Ancodontids have a tall skull with high-positioned orbits, enlarged as well as tusk-like canines and incisors. Ruminantids bear antlers or horns that are often large and complex in shape. The mastoid bone is exposed between the squamosal and occipital bones. Cetaceans have a highly modified skull caused by posterior migration of the nostrils. The premaxilla and maxilla bones form the roof of the rostrum. Enlarged occipital bones occupy the posterior part of the skull. The nasal and parietal bones are highly reduced in size	[147–157]
*Euarchontoglires*		
Scandentia	Treeshrews have a unique, prominent hole in the zygomatic arch. The postorbital bar is well developed and contacts the zygomatic arch. There is variation in skull morphology within *Tupaia glis* that might be due to the geographic barriers between populations. For example, island populations have a smaller skull than continental ones	[158–160]
Rodentia	Rodent skulls are unique, bearing a single pair of persistently growing incisors in the upper and lower jaws. The orbital cavity is located dorsal to the cheek teeth. The zygomatic arch fuses to the maxilla in line with the first cheek teeth. The vertical ramus of the dentary bone is enlarged and provides the area for insertion of the masseter muscle. Rodentia consists of three suborders: Myomorpha, Sciuromorpha, Hystricomorpha. Myomorpha have enlarged temporal bones where a large temporal muscle attaches. The muscle produces high mastication power using cheek teeth. Sciuromorpha have a large vertical ramus of dentary bone where the masseter muscle attaches. This produces a high power in biting using incisors. Hystricomorpha have a large infraorbital foramen in their skull. Both phylogenetic and ecological factors influence the determination of skull morphology in rodents. In Hystricomorphids (e.g., guinea pigs, porcupines, and spiny rats), phylogenetic constraints are more important than ecological factors in generating morphological variation of the dentary bone. On the other hand, morphological variation of skulls is mainly brought about by ecological factors. Hystricomorphids living in open habitats, such as guinea pigs, have upward-facing orbits and a wide basicranium. Hystricomorphids living in woody areas, such as spiny rats, have more laterally facing orbits and a narrow basicranium	[161–194]
Lagomorpha	Rabbits have a fenestrated skull which is unique among mammals. The fenestration (lattice-like bone) is seen in the proximolateral part of the rostrum. Morphological disparity of skull morphology in the family Leporidae is mainly explained by differences in the degree of facial tilt among species	[195–198]
Primates	Skull morphology is very different between haplorhines and strepsirrhines, mainly in relative skull length and width and facial depth. Haplorhines tend to have a mediolaterally wide as well as dorsoventrally tall skull. Strepsirrhines have a narrower, shallower skull, an elongated face, and a narrower snout. Intraspecific variation in skull shape has been studied in several groups of primates, including Cercopithecoidea and Hominidae	[199–214]
Dermoptera	The two extant colugo species have skulls with large front-facing orbits that improve binocular vision. The position of three pairs of upper incisors is shifted laterally, and the second upper incisors are transformed into a canine-like shape. The first two lower incisors are broad and form a comb-like shape	None

However, the genetic and developmental mechanisms underlying the diversification of mammalian craniofacial morphology remain largely unknown. In this review, we compiled the recent findings in the developmental genetics of mice, a model mammalian species, to attempt to deduce the potential diversification mechanisms of mammalian facial morphology. We also introduce the results of previous studies in which a strong correlation between the number of nucleotide tandem repeats within the *Runx2* gene and the facial length in some placental mammals was reported. Finally, we focus on bats (order Chiroptera), which display a substantial degree of craniofacial diversity and discuss their potential as a model for understanding the evolution of mammalian craniofacial morphology.

## Molecular and cellular mechanisms creating diversity in facial morphology uncovered by mouse transgenesis

Mouse transgenesis is a powerful tool to infer the function of genes related to vertebrate morphogenesis. We examine the phenotypes of transgenic mice to gain insights into the molecular and cellular mechanisms that produce morphological variation in mammalian faces. We focused on two developmental events: (1) growth and patterning of the facial primordium and (2) ossification of the facial bones that lead to a shortened face and the orofacial cleft (Table 2).

**Table 2:** The genes involved in shortening the face and making the orofacial cleft in mouse

Gene	Mutant	Phenotype	Protein function	Signaling pathway	References
*Bmp2*/*Bmp4*	*Wnt1Cre; Bmp2* ^*f/f*^ *; Bmp4* ^*f/f*^	Truncated face	Signaling molecule	BMP	[30]
*Bmpr1a*	*NestinCre; Bmpr1a* ^−*/f*^	CL/P	Receptor	BMP	[215]
*Ctnnb1*	*Ctnnb1* ^*f/f*^ *; Crect*	Truncated face	Transcription factor; regulation of cell–cell adhesion	Wnt	[22]
*Msx1*	*Msx1* ^−*/*−^	Truncated face	Transcription factor	BMP Shh	[23]
*Ptch1*	*Wnt1Cre; Ptch1* ^*c/c*^	CL	Receptor	Hh	[216]
*Smo*	*Wnt1Cre;Smo* ^−*/c*^	Truncated face	Receptor	Hh	[217]
*Tfap2a*	*Tfap2a* Neo(hypomrpha)/Null model	CL/P	Transcription factor	FGF Notch BMP	[218]
*Wnt3*	*Wnt3* ^−/−^	CL/P	Signaling molecule	Wnt	[219]
*Wnt9b*	*Wnt9b* ^−^ */clf1*	CL/P	Signaling molecule	Wnt	[220]
*Wnt5a*	*Wnt5a* ^−*/*−^	Truncated face	Signaling molecule	Wnt	[25, 31, 32]
*Bmpr1a*	*Osr2*-*IresCre;Bmpr1a*^*f/f*^	SMCP	Receptor	BMP	[38]
*Fblin5*	*Fblin5* ^−*/*−^	Shortened face	Secreted extracellular matrix protein	MAPK-Erk	[35]
*Fgf8*	*Osr2cre;Rosa26R*-*Fgf8*	CP	Growth factor	FGF	[221]
*Kif3a*	*Wnt1Cre;Kif3a* ^*f/f*^	CP	Motor protein	Hh	[222]
*Msx1*	*Msx1* ^−*/*−^	Shortened face CP	Transcription factor	BMP Shh	[223, 224]
*Mn1*	*Mn1* ^−*/*−^	Shortened face CP	Transcription coregulator	N/A	[225]
*Shox2*	*Shox2* ^−*/*−^	CP	Transcription factor	N/A	[226]
*Tbx22*	*Tbx22* ^−*/*−^	SMCP	Transcription factor	BMP FGF	[39]
*Tgfβr2*	*Wnt1Cre; Tgfβr2* ^*f/f*^	CP	Receptor	TGF-β	[227]

### Growth and patterning of the facial primordium

Formation of mammalian faces begins at the pharyngula stage of embryogenesis, through growth and fusion of the five facial processes: the frontonasal process (FNP), medial nasal processes (MNPs), lateral nasal processes (LNPs), maxillary processes (MAXs), and mandibular processes (MANs) [19]. In the facial development of mice, FNP first expands anteriorly in a nine-day-old embryo (E9.0). Subsequently, MNPs and LNPs start to bulge out from the FNP at E10.0. These two processes surround the nasal placodes, MNP surrounds its medial aspect, and LNP surrounds its lateral aspect. During the same embryonic stage, MAXs begin to bulge anteriorly covering the ventrolateral aspect of the FNP. MAXs and the FNP continue to grow and fuse to each other in later stages to form the upper jaw. Paired MANs begin to grow anteriorly at E9.0 and fuse to one another at the midline to form the mandible [19, 20].

The early patterning of the mammalian face is regulated by migration and proliferation of the neural crest-derived mesenchyme (ectomesenchyme hereafter) [19, 21]. Mice with genetic defects related to the migration or proliferation of the ectomesenchyme possess a shortened face [22–25] and/or cleft lip (CL) occasionally accompanying the cleft palate (CP) [19, 26–28].

Several major signaling pathways, including BMP, FGF, Shh, and Wnt signaling pathways, are associated with outgrowth and fusion of the facial processes [19]. Repression of the up-stream component genes of these signaling pathways (e.g., *Bmp4*, *Fgf8*, *Shh*, and *Wnt3*) leads to a truncated face [19, 22, 24, 29, 30]. Recent papers have reported that migration of ectomesenchyme in the heads of mouse embryos are directly regulated by *Wnt5a*, a ligand of non-canonical Wnt signaling pathway [22, 25, 31, 32]. Alteration of the level of neural crest-specific *Wnt5a* expression (by both knockout and over-expression) results in a widened, shortened face [25, 33]. In *Wnt5a* conditional knockout mice, the migration pattern of the ectomesenchyme that later occupies the internal space of the facial processes is altered from that in control wild type mice [25]. The change in the ectomesenchyme migration pattern was attributed to the disruption of the directionality of cell division [25]. The induction of the internal facial structures (e.g., cartilage, bones, sensory compartments, muscles, glands, and teeth) was not influenced, and the lower jaw’s volume in the *Wnt5a* conditional knockout mouse was almost equivalent to that of the control mouse [25]. These results suggest that *Wnt5a* could play a crucial role in generating a shortened, widened face (truncated face) as naturally seen in koalas, sloths, the great apes, and cats through regulating the ectomesenchyme’s migration pattern, which in turn governs growth and organization of the facial processes (Fig. 1).

Disruptions in the growth and fusion of the facial processes also cause CL with or without CP (collectively called ‘CL/P’) [26–28]. A fusion of the facial processes first occurs between LNP and MNP, followed by a fusion of LNP and MAX. Finally, the anterior ends of both MAX and MNP are fused to one another. Fusion of the facial processes is initiated by contact of the epithelium of each facial process through proper organization of the facial processes [19]. Subsequently, the epithelial seam between coadjacent facial processes disappears due to apoptosis. Fusion of the MNP and the MAX and fusion of the MNP and the LNP are defective in mutants of the genes (e.g., *Bmp4*, *Bmpr1a*, *Tcfap2a*, *Sox11*, and *Wnt9b*) that regulate apoptosis within the epithelium as well as outgrowth and organization of the facial processes. Failure of these facial processes fusing accompanies CL/P [26].

### Ossification of the facial bones

The palate of mammals separates the oral cavity from the nasal cavity and is subdivided into the anterior bony hard palate (palatal bones) and posterior soft palate [34]. The formation of the palate (palatogenesis) proceeds in two steps, the primary and secondary palate formations. In mouse development, the primary palate is formed by the fusion of the MAXs and MNPs at E11.5. Subsequently, the secondary palate is formed through three consecutive events. First, a pair of palatal shelves is formed by an uplift of the tongue at E11.5. Second, at E14.5, each palatal shelf grows medially above the tongue through ‘palatal shelf elevation’ [34]. Third, the left and right palatal shelves meet and fuse at the midline at E15.0 with fusion completing at E17.0. Palatal bones (anterior premaxilla derived from the ectomesenchyme of the primary palate, and central maxilla and posterior palatine that are derived from the ectomesenchyme of the secondary palate) begin to form at E14.5.

In contrast to defects in facial process development that produce an extremely shortened face (see the previous section), defects in facial bone formation, which occur in later phases of facial development, lead to a shortened face with milder dysmorphology. For example, *Fbln5* knockout mice exhibit decreased outgrowth of the premaxilla bones during postnatal stages, compared to control wild type mice [35]. Fibulin-5 is an extracellular matrix protein deposited as a fibrous matrix in neural crest-derived craniofacial suture mesenchyme and plays a role as a regulator of cellular function such as cell proliferation [35, 36]. While premaxilla-maxilla suture mesenchyme in *Fbln5* knockout mice were capable of differentiating into osteoblasts, suture cells in the mutant were less proliferative, suggesting fibulin-5 is indispensable for the regulation of facial suture mesenchymal cell proliferation required for craniofacial skeletal morphogenesis [35]. External facial morphology of adult *Fbln5* knockout mice is almost normal, although facial length is slightly shortened compared to the control [35].

Defected facial bone development also leads to a submucous cleft palate (SMCP). SMCP is a clinical subgroup of CP. While CP is characterized by the whole palate (including both bones and epithelium) separated at the midline, SMCP is characterized by incomplete fusion of left and right palatal bones at the midline without cleft formation in the oral epithelium covering the bones. In mouse transgenesis, SMCP is only observed in the region between left and right maxilla bones. Only two genes that cause SMCP have been reported to date, *Bmpr1a* and *Tbx22*. In *Osr2*-*IresCre;Bmpr1a*^*f/f*^ transgenic mice, *Bmpr1a* was specifically knocked out in the tissue constructing the secondary palate. *Osr2*, whose promoter sequence was used for tissue/time-specific *Bmpr1a* knockout, is uniquely expressed in secondary palate morphogenesis in mice (see [37] for detail). The tissue-specific inactivation of *Bmpr1a* causes reduction of mesenchymal condensation in the anterior part of the secondary palate which subsequently differentiates into the maxilla bones [38]. Expression of *Runx2*, *Osterix*, and *Dlx5*, genes encoding transcriptional factors for bone development, is severely down-regulated in the anteromedial part of the secondary palate of *Osr2*-*IresCre;Bmpr1a*^*f/f*^ transgenic mice. As a result, elongation of the maxilla bones toward the midline is blocked, resulting in a cleft between the left and right maxilla bones [38]. Tbx22 is a transcription factor required for palatal bone formation [39]. *Tbx22* knockout embryos bear a CP or SMCP accompanied by delayed osteoblast differentiation and hypotrophic maxilla bones [39].

To our knowledge, elongation of the face in transgenic mice compared to wild type mice has not been reported to date. In fish and birds, longer and more pointed jaws or beaks are formed by up-regulation of calmodulin signaling [40–43]. In mammals, however, the function of calmodulin signaling in facial development is poorly understood. *Runx2* may regulate facial length in mammals. We briefly review the correlation between facial length and the variation of glutamine/alanine tandem repeats within Runx2 in the next section.

## The number of *Runx2* tandem repeats and mammalian facial length

There are long- and short-faced taxa in each mammalian group, and both face types show a high degree of diversity and evolvability in facial length (Fig. 1). Runx2 (Runt-related transcription factor 2) is an important transcription factor protein that plays multiple roles in bone development (e.g., osteoblast differentiation) in vertebrates including mammals [44–46] (reviewed in [47]). Runx2 enhances early osteoblast differentiation but inhibits terminal osteoblast differentiation [48]. Therefore, up-regulation of *Runx2* leads to accelerated (via early onset of osteoblast differentiation) and extended (via delayed termination of osteoblast differentiation) bone development, while down-regulation of *Runx2* results in delayed, shortened bone development [48, 49].

The Runx2 protein contains a highly conserved RUNT DNA binding domain and a repetitive glutamine (Q) and alanine (A) domain [46, 50]. Changes to the tandem repeat glutamines to alanines ratio (QA ratio), calculated by dividing the number of consecutive glutamines by the number of consecutive alanines within Runx2, alter transcriptional activity of Runx2 and its target genes [49, 51].

**Fig. 2:**
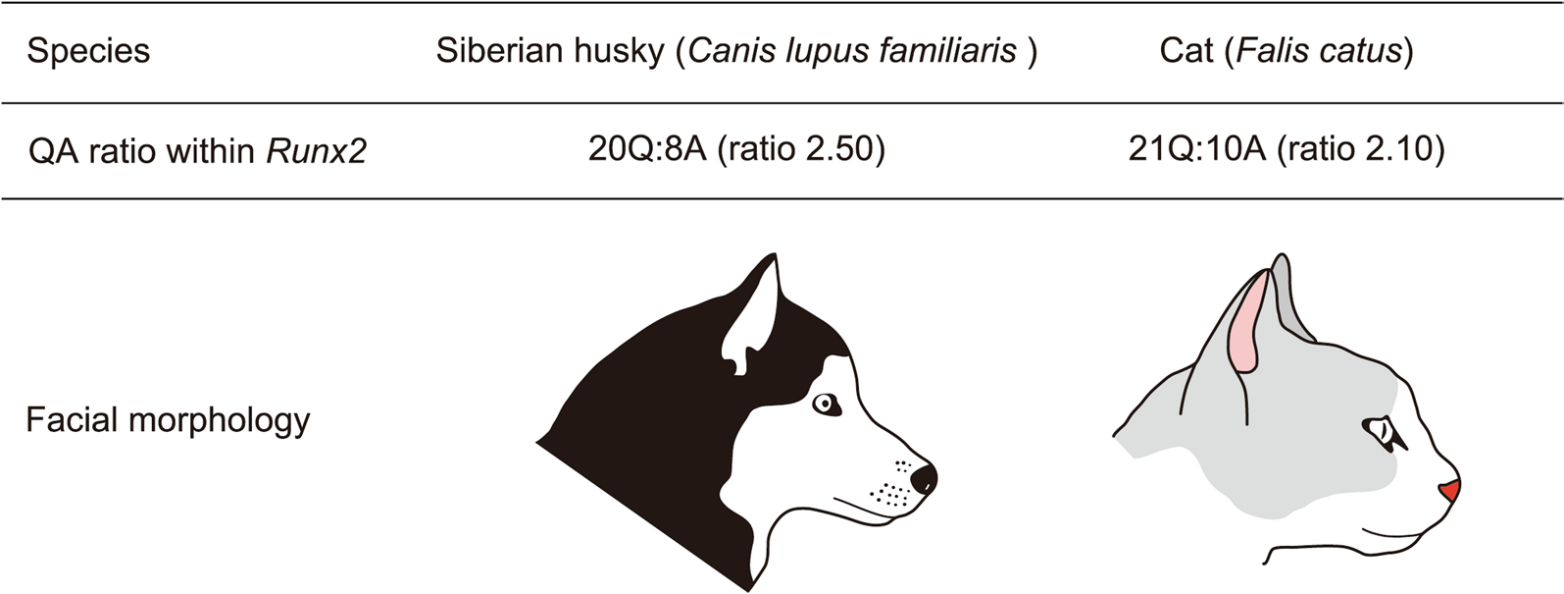
Correlation between QA ratio of the transcription factor Runx2 and facial length in order Carnivora. The Siberian husky, a breed of the domestic dog (*Canis lupus familiaris*), has 20 glutamine- and 8 alanine-coding nucleotide sequences within the repetitive glutamine and alanine domain of *Runx2*. QA ratio, calculated by dividing total glutamine-coding sequences by total alanine-coding sequences, is 2.50 and results in a longer face. The domestic cat (*Falis catus*) has 21 glutamine- and 10 alanine-coding nucleotide sequences within the corresponding domain of *Runx2*. QA ratio is 2.10 and results in a shorter face

The Runx2 QA tandem repeat ratio is correlated with facial length variation in carnivorans [49, 52, 53]. Species with higher QA ratios have longer faces [49] (Fig. 2). In contrast, a lower QA ratio leads to lower transcriptional activity of *Runx2* and results in short-faced carnivorans [49] (Fig. 2). This suggests that the QA ratio is associated with allometric variation in carnivoran facial length and the timing of facial bone (e.g., premaxilla, maxilla, nasal, jugal, vomer, palatine, and dentary) ossification. A similar pattern has been reported in primates [54].

Conversely, there is no correlation between the Runx2 QA tandem repeat ratio and facial length in xenarthrans and afrotherians [55], and marsupials [51]. Although marsupials display variation in facial length roughly equivalent to that observed in placentals (Fig. 1), almost no variation is observed in the nucleotide sequence of glutamine/alanine repeats in *Runx2* [51]. The extreme conservation of nucleotide sequence and the QA ratio in marsupials may heavily constrain the timing of facial bone ossification in marsupial species [51]. These results suggest that the variations of facial length in xenarthrans, afrotherians, and marsupials are brought about by distinct molecular mechanisms. For example, a missense mutation in the gene *Bmp3* (that encodes a growth factor, Bone morphogenetic protein 3) causes brachycephaly (shortened head) in domestic dogs [56]. We recommend further research concerning the role of morphogenetic genes such as *Bmp3* to improve our understanding of the mechanisms generating facial length variation in mammals other than carnivorans and primates.

## Bats: a model for understanding the diversification of mammalian craniofacial morphology

As reviewed in section II, our understanding of mammalian facial development mechanisms has been informed by studies of laboratory mice. However, the developmental mechanisms that produce facial morphology in non-model, wild mammal species have been only partially understood, perhaps due to difficulties in obtaining embryonic materials for analyses. More is understood about the molecular and cellular mechanisms underlying diversification of facial (beak) morphology in non-model bird species thanks to a series of evo-devo studies of Darwin’s finches, one of the most famous examples of adaptive radiations in vertebrates [40, 57–62]. Although model mammals help us to understand the basic mechanisms of mammalian morphogenesis, studying non-model species is necessary to identify other molecular and cellular mechanisms that lead to the morphological evolution of this group of vertebrates (including humans). Here, we focus on bats as a potential model for understanding evolution of mammalian craniofacial morphology.

**Fig. 3:**
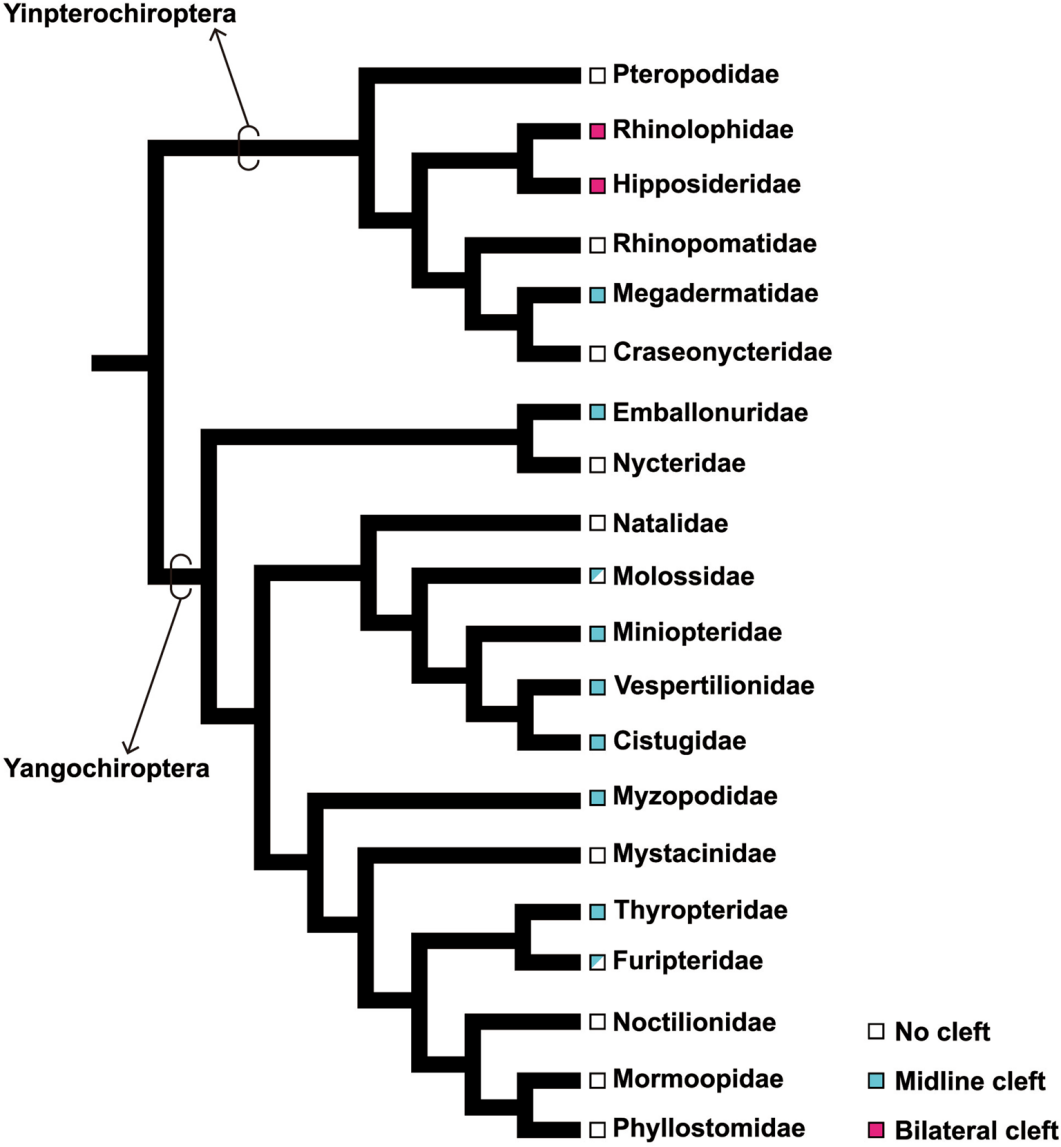
Phylogeny of bats and evolution of orofacial cleft. The basic framework of chiropteran phylogeny is based on Teeling et al. [65]. Phylogenetic relationships in the superfamily Vespertilionoidea (Natalidae, Molossidae, Miniopteridae, Vespertilionidae, and Cistugidae) adapted from Lack et al. [228]. The midline cleft is possessed by nine different families of bats. In Molossidae, at least two genera (*Mormopterus*, and *Tadarida*) bear the midline cleft. In Furipteridae, only the genus *Furipterus* bears the midline cleft. The bilateral cleft evolved only once in the common ancestor of Rhinolophidae and Hipposideridae. Character mapping was based on Orr et al. [88]

Bats (order Chiroptera) are the second largest group of mammals after rodents [2, 63]. More than 1300 extant bat species are known, classified into 20 families [63]. Recent molecular phylogenetic studies [64–67] identified two major clades within bats, the Yinpterochiroptera and Yangochiroptera (Fig. 3). Chiropterans are distributed worldwide in all but the coldest regions [63], probably facilitated by the evolution of flight [68–80].

**Fig. 4:**
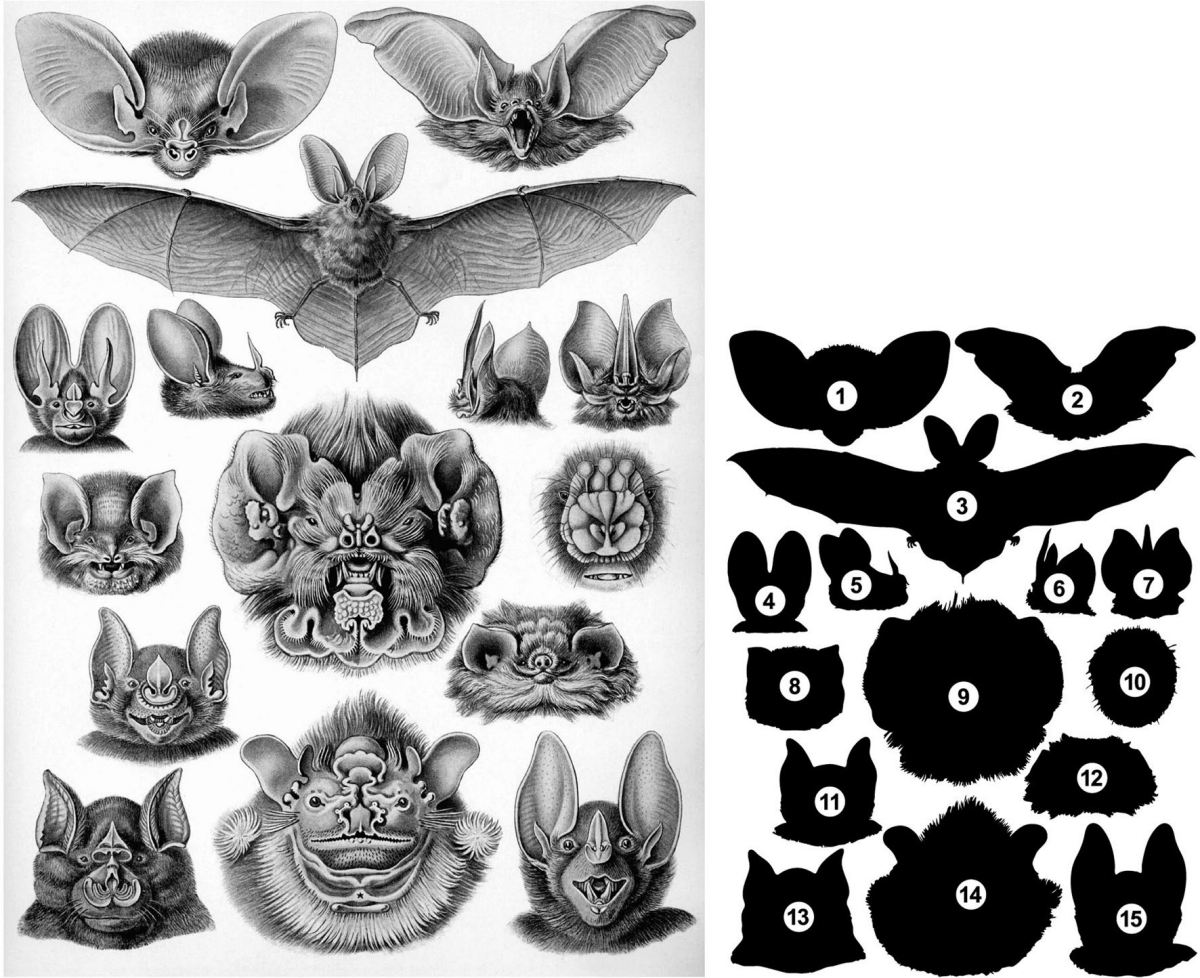
Diversity of craniofacial morphology in bats. Left, a picture drawn by Ernst Haeckel, an influential comparative embryologist and artist [81]. Right, the silhouettes of the bat species illustrated in the Haeckel’s picture: (*1*) lesser long-eared bat (*Nyctophilus geoffroyi*), frontal view of the head; (*2*) brown long-eared bat (*Plecotus auratus*), frontal view of the head; (*3*) brown long-eared bat, entire body; (*4*) lesser false vampire bat (*Megaderma spasma*), frontal view of the head; (*5*) big-eared woolly bat (*Chrotopterus auritus*), lateral view of the head; (*6*) Tomes’s sword-nosed bat (*Lonchorhina aurita*), caudo-lateral view of the head; (*7*) Tomes’s sword-nosed bat, frontal view of the head; (*8*) Mexican funnel-eared bat (*Natalus stramineus*), frontal view of the head; (*9*) Antillean ghost-faced bat (*Mormoops blainvillei*), frontal view of the head; (*10*) flower-faced bat (*Anthops ornatus*), high magnification of noseleaf; (*11*) greater spear-nosed bat (*Phyllostomus hastatus*), frontal view of the head; (*12*) thumbless bat (*Furipterus horrens*), frontal view of the head; (*13*) greater horseshoe bat (*Rhinolophus ferrumequinum*), frontal view of the head; (*14*) wrinkle-faced bat (*Centurio senex*), frontal view of the head; (I) spectral bat (*Vampyrum spectrum*), frontal view of the head

Although largely neglected by biologists, diversity in bat facial morphology is astonishing. This diversity reflects their adaption to various environments and highly impressed Ernst Haeckel, an influential comparative embryologist and an artist in nineteenth century [81] (Fig. 4). New World leaf-nosed bats (family Phyllostomidae) are especially known for their incredible facial diversity [82, 83]. Phyllostmid facial length is strongly correlated with diet [84–86]. For example, frugivorous species (e.g., the wrinkle-faced bat, *Centurio senex*) have a truncated, widened face that exerts a high bite force. In contrast, nectarivorous species (e.g., the mexican long-tongued bat, *Choeronycteris mexicana*) have a long, narrow face that helps them to insert their rostrum into flowers. However, the molecular and cellular mechanisms that regulate the facial length of bats and are responsible for generating existing diversity in craniofacial morphology are poorly understood.

Bats have a unique morphological feature in the rostral part of the upper jaw, an orofacial cleft on the premaxilla and maxilla bones that is anatomically similar to that observed in humans with congenital anomalies [87, 88]. There are two types of chiropteran orofacial cleft, midline and bilateral clefts. The midline cleft is observed in nine families of bats: Megadermatidae, Emballonuridae, Molossidae, Miniopteridae, Vespertilliionidae, Cistugidae, Myzopodidae, Thyropteridae, Furipteridae [88] (Fig. 3). Midline clefts are U-shaped clefts present between two premaxilla bones that are highly reduced in size (Fig. 6). Each premaxilla bone bears two permanent incisors and is completely fused to the maxilla bone posteriorly. The inner space of the cleft is occupied with a robust, translucent, fibrous membrane. The bilateral cleft is only seen in Rhinolophidae and Hipposideridae [88] (Fig. 3). In this cleft type, the premaxilla bone, which bears a single diminutive incisor, is separated from the laterally located maxilla bone by a cleft. The cleft is filled with fibrous connective tissue. The posterior margin of the medially fused premaxilla bones is loosely connected to the maxilla bones with fibrous connective tissue.

Bat orofacial clefts may contribute to reduction of returning echolocation signal interference, modulation of nasal acoustic emissions, increasing oral gape to facilitate capture of large prey, reduction of overall weight, and increase of olfactory ability [88]. However, the molecular and cellular mechanisms underlying orofacial cleft development in bats and the degree to which development of the two cleft types is similar are currently unknown.

Few studies have investigated the molecular mechanisms related to craniofacial diversity in bats. One such study by Phillips et al. [89] focused on Pax9, a transcription factor that plays an important role in vertebrate craniofacial and dental development. The authors compared nucleotide sequences of the 3′ untranslated region (UTR) of *Pax9* among phyllostomids, vespertilionids, and other mammalian orders and identified four Musashi-binding elements (MBE) within conserved regions of the 3′ UTR [89]. The number of MBEs in morphologically diverse phyllostomid bats varied but was invariant in morphologically similar vespertilionid bats with the exception of a *Murina* species [89]. Because the number of MBEs may affect the expression level of *Pax9*, the authors proposed that the evolution of Pax9 regulation may be a contributing mechanism to the radiation of craniofacial morphological diversity in bats [89]. Although this study provides valuable insight into a potential genetic mechanism underlying the evolution and diversification of craniofacial morphology in phyllostomid bats, our understanding of the fundamental facial development mechanisms is far from complete.

Because convergence or parallel evolution of morphological traits in vertebrates is often brought about by identical genetic mechanisms (e.g., [90–93]), common mechanisms might regulate facial length even in bats (superorder Laurasiatheria) and rodents (superorder Euarchontoglires; Table 2).

**Fig. 5:**
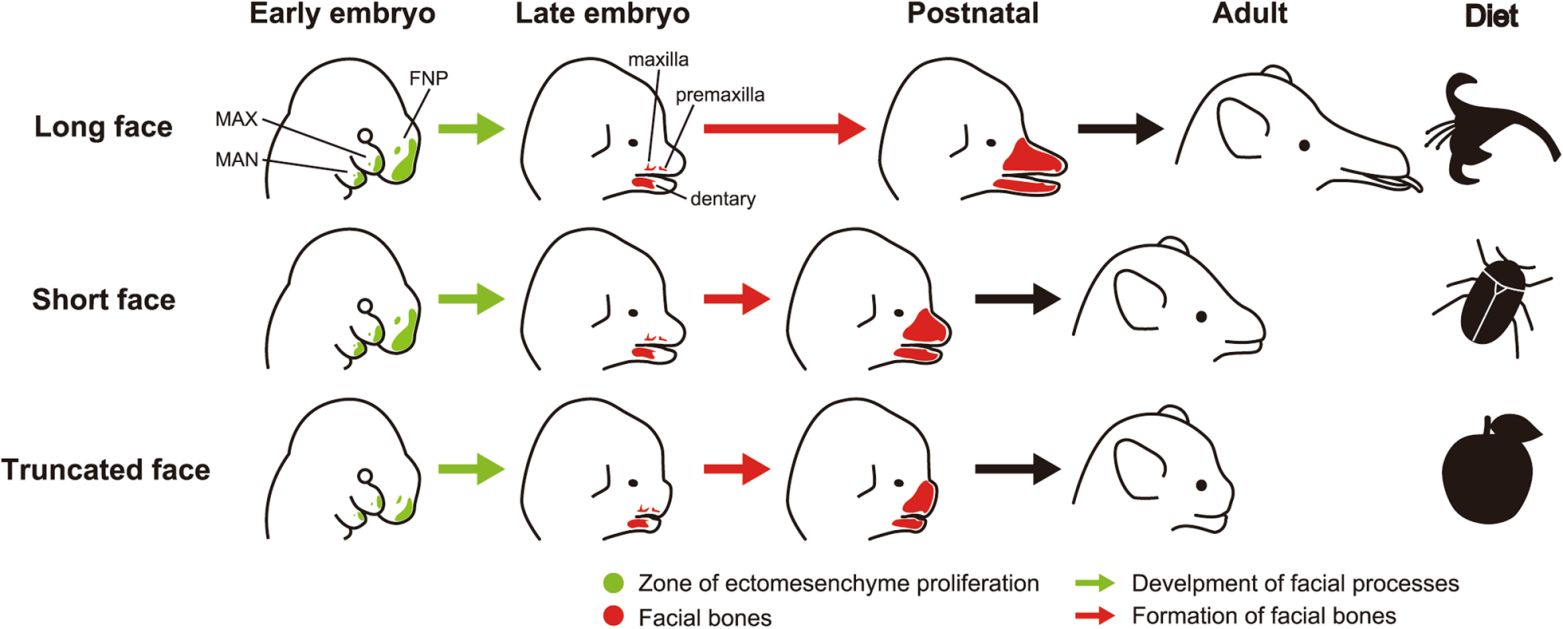
Schematic diagram depicting potential developmental mechanisms regulating facial length in bats. Top row: A long face possessed by some nectarivorous bat species (e.g., *Choeronycteris mexicana*) is formed through extension of the period of facial bone (e.g., premaxilla, maxilla, and dentary) development. Middle row: A short face possessed by many insectivorous or omnivorous bat species (e.g., *Macrophyllum macrophyllum*) is formed through shortening the period of facial bone development. Bottom row: A truncated face possessed by some frugivorous bat species (e.g., *Centurio senex*) is formed through deficient outgrowth of the facial processes in pharyngula stages. This could be attributed to reduced proliferation as well as disrupted migration of cranial neural crest cells (ectomesenchyme) occupying the internal space of the facial processes. FNP, frontonasal process; MAN, mandibular process; MAX, maxillary process

In mice, a shortened face without apparent facial bone defects is mainly brought about by a decrease in proliferation and differentiation of the ectomesenchyme which later differentiates into osteoblasts [35]. In addition, facial length variation observed in carnivorans and primates are correlated with the level of activity of *Runx2*, which influences facial bone development duration [49]. Therefore, facial length variation in bats could be attributed to differences in the duration of facial bone development among species. For example, nectarivorous bats (e.g., *Choeronycteris mexicana*) have a relatively longer face. In this case, the duration of facial bone development might be extended, giving facial bones time to enlarged, especially anteriorly (Fig. 5). Conversely, insectivorous or omnivorous bats (e.g., *Macrophyllum macrophyllum*) have a relatively shorter face. Here, the period of facial bone development may be shortened leading to earlier completion of facial bone growth and preventing further anterior elongation (Fig. 5). Indeed, heterochronic shift in formation and growth of the palatal bones may produce variations of craniofacial morphology in phyllostomid bats [94]. Sears supposed that the diversity of palate shapes along phyllostomids is the result of relatively subtle evolutionary changes in later rather than earlier developmental event. Although it is likely that *Runx2* plays a crucial role in producing facial length diversity in carnivorans and primates [49, 52–54], its function in chiropteran craniofacial development has yet to be identified and warrants further investigations.

The truncated face of *Wnt5a* conditional knockout mice is brought about by the disruption of ectomesenchyme migration within the facial processes [25]. Notably, some phyllostomid bats (e.g., *Centurio senex*) possess an extremely truncated face that shares multiple characteristics with *Wnt5a* knockout mice faces. Therefore, facial morphology in these bat species might be derived from changes in expression of the genes that control direction of migration of the ectomesenchyme through regulating the directionality of cell division within the facial processes (Fig. 5). It would be interesting to compare Wnt5a activity and expression pattern in facial ectomesenchyme among chiropteran species.

**Fig. 6:**
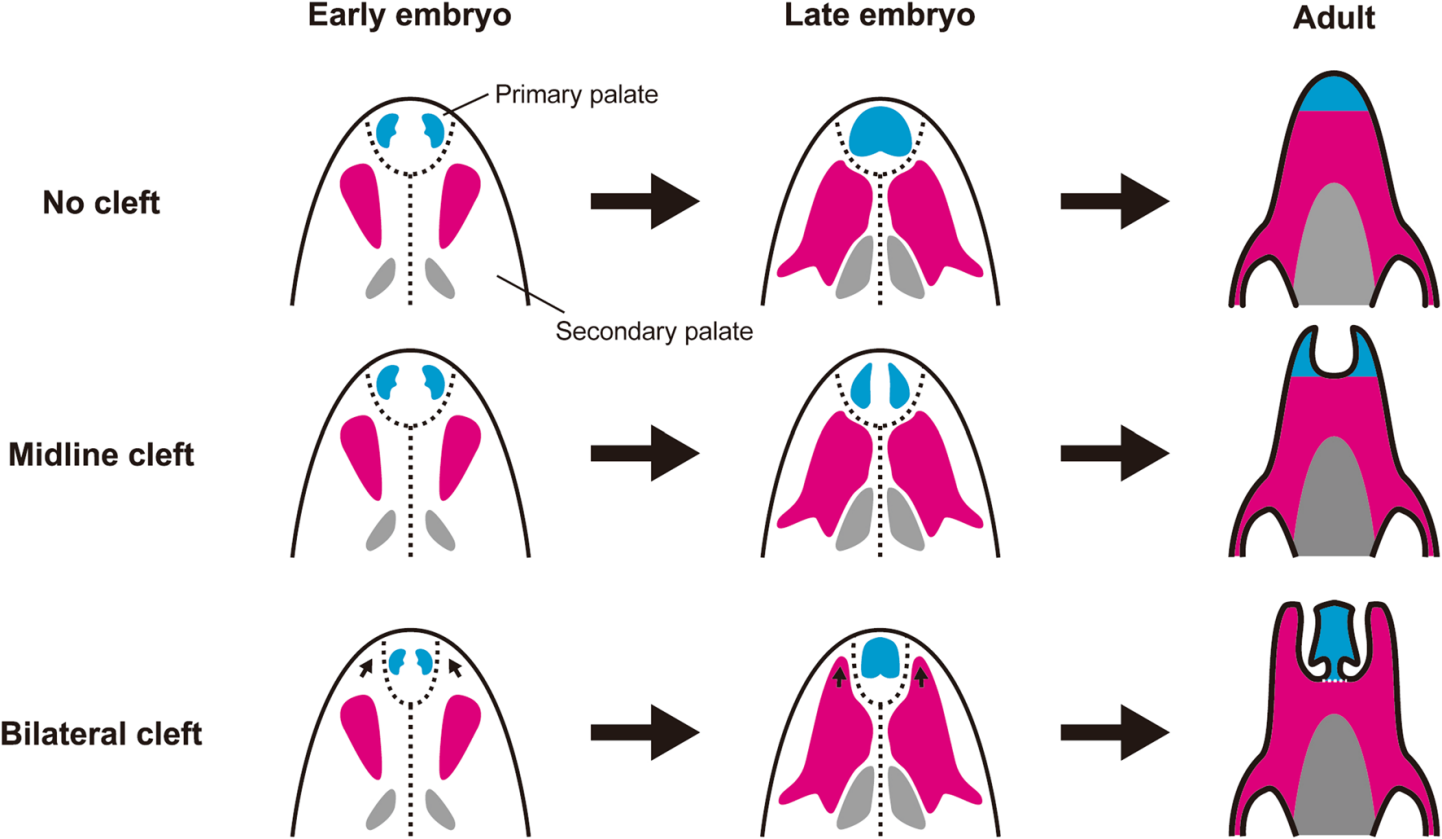
Schematic diagram depicting potential developmental mechanisms forming orofacial clefts in bats. Top row: Most bat species do not have an orofacial cleft in their skull. Palatal bones are composed of three sets of bones: the premaxilla (blue), the maxilla (pink), and the palatine (gray). Each is a paired structure originally, having left and right elements. In adults, these bones are fused to each other and form the palate. In bats, the sutures between the bones constructing their skull (including the palate region) usually become indistinct through complete fusion of the bones. Middle row: In bat species with the midline cleft, the growth of the premaxilla bones toward the midline is inhibited and this makes medially unfused premaxilla bones. Heterochrony in ossification of the premaxilla bone (shorter and/or delayed ossification of the bone compared to the ancestor) may result in such a small-scale morphological change in the tip of the face. Bottom row: In bat species with the bilateral cleft, the cleft is likely formed through three developmental steps: (1) the domain of the secondary palate expands antero-medially, possibly through changes in growth and patterning of the facial processes at the early stages of embryogenesis. This narrows the space for the primary palate (arrows in the left illustration). (2) The maxilla bones are elongated anteriorly (arrows in the central illustration) compared to in bats species without orofacial clefts as well as those with midline clefts, acquiring its anterior projection. Simultaneously, the position of the premaxilla bones is confined at the center of the tip of the face, due to reduction of the space for its lateral expansion. (3) The boundary between the (anterior) premaxilla and (posterior) maxilla is left as a joint connected through loose connective tissue (a white dashed line in the right illustration). The space between the (medial) premaxilla and the (lateral) maxilla bones is left as a cleft

The orofacial clefts observed in bats are morphologically categorized as SMCP. They are probably brought about by changes in premaxilla and maxilla bone formation. As we introduced in section II, *Osr2*-*IresCre;Bmpr1a*^*f/f*^ mice have a cleft between paired maxilla bones [38]. If *Bmpr1a* expression is specifically inactivated in the primary palate region using a similar transgenic technique (e.g., using a promoter of the gene that is uniquely expressed in the primary palate in gene knockout), a cleft may appear between paired premaxilla bones that are derived from the ectomesenchyme distributed within the primary palate. Considering this, the midline cleft in bats, which is present between two premaxilla bones, could be explained by domain-specific repression or down-regulation of *Bmpr1a* in the ectomesenchyme within the primary palate (instead of the secondary palate) that later gives rise to the premaxilla bones (Fig. 6). Because Bmpr1a is a receptor of the growth factor, the down-regulation of *Bmpr1a* may decrease the degree of ossification of the premaxilla bone through heterochrony (shorter and/or delayed ossification of the bone compared to the ancestor) and may result in such a small-scale morphological change in the tip of the face.

The formation of the bilateral cleft could be much more complicated, perhaps associated with extensive alterations of the developmental program. The premaxilla bones are derived from the ectomesenchyme distributed within the primordium of the primary palate, while the maxilla bones are derived from that of the secondary palate. Therefore, in the facial development of bat species bearing the bilateral cleft, the relative position of the primary and secondary palates might be changed through alterations in formation and organization of the facial processes from those in bat species without orofacial cleft. We speculate that the bilateral cleft developed through the following three steps (Fig. 6). First, the ectomesenchyme occupying the secondary palate expanded its distribution antero-medially and restricted the space for primary palate development at the tip of the face. Second, the osteoblasts derived from the ectomesenchyme distributed within the anterior part of the secondary palate differentiated into bone and made anterior projection of the maxilla bones surrounding the premaxilla bone laterally. Thus, the position of the premaxilla bone became restricted at the center of the tip of the face. Third, inhibition of ossification at the suture between the medially positioned premaxilla and laterally positioned maxilla bones left the unossified area between the two bones as a cleft.

Orofacial clefts occur as a craniofacial anomaly in humans at a relatively high frequency (approximately 1 in 700 live births) [88]. Investigating the mechanisms behind orofacial cleft formation in bats may contribute not only to understanding the reason why this cranial feature, which usually occurs as a skeletal pathology in other mammals groups including humans, appears as a normal phenotype in bats, but also to developing novel therapies against human orofacial cleft.

In the last 15 years, several studies have described in detail the overall embryonic development [95–100] and specifically wing development of bat species where embryos could be obtained [68–77, 79, 80]. We believe that examination of bat facial development and its comparisons among the species provide profound insights into the molecular and cellular bases of craniofacial morphology diversification in mammals.

## Conclusions

In this paper, we have reviewed recent advances in understanding how mammalian faces are formed and discussed how these data are being applied to make new hypotheses about the diversity creation in mammalian craniofacial morphology. Small-scale changes in facial morphology from the ancestor, such slight changes in facial length and the evolution of the midline cleft in some lineages of bats could be attributed to heterochrony in facial bone ossification. In contrast, large-scale changes in facial morphology from the ancestor, such as a truncated, widened faces, as well as the evolution of the bilateral cleft in some bat species, could be brought about by changes in growth and patterning of the facial primordium (the facial processes) at the early stages of embryogenesis. Significant work remains to be done to test these hypotheses.

## Abbreviations

CL: cleft lip
CP: cleft palate
FNP: frontonasal process
LNP: lateral nasal process
MAN: mandibular process
MAX: maxillary process
MBE: Musashi-binding elements
MNP: medial nasal process
SMCP: submucous cleft palate

## References

[1] Kemp TS (2005). The origin and evolution of mammals.

[2] Wilson DE, Reeder DM (2005). Mammal species of the world.

[3] Vaughan TA, Ryan JM, Czaplewski NJ (2013). Mammalogy.

[4] Feldhamer GA, Drickamer LC, Vessey SH, Merritt JF, Krajewski C (2014). Mammalogy: adaptation, diversity, ecology.

[5] Meredith RW, Janečka JE, Gatesy J, Ryder OA, Fisher CA, Teeling EC, Goodbla A, Eizirik E, Simão TL, Stadler T, Rabosky DL, Honeycutt RL, Flynn JJ, Ingram CM, Steiner C, Williams TL, Robinson TJ, Burk-Herrick A, Westerman M, Ayoub NA, Springer MS, Murphy WJ (2011). Impacts of the cretaceous terrestrial revolution and KPg extinction on mammal diversification. Science.

[6] Dos Reis M, Inoue J, Hasegawa M, Asher RJ, Donoghue PC, Yang Z (2012). Phylogenomic datasets provide both precision and accuracy in estimating the timescale of placental mammal phylogeny. Proc Biol Sci.

[7] Song S, Liu L, Edwards SV, Wu S (2012). Resolving conflict in eutherian mammal phylogeny using phylogenomics and the multispecies coalescent model. Proc Nat Acad Sci.

[8] O’Leary MA, Bloch JI, Flynn JJ, Gaudin TJ, Giallombardo A, Giannini NP, Goldberg SL, Kraatz BP, Luo ZX, Meng J, Ni X, Novacek MJ, Perini FA, Randall ZS, Rougier GW, Sargis EJ, Silcox MT, Simmons NB, Spaulding M, Velazco PM, Weksler M, Wible JR, Cirranello AL (2013). The placental mammal ancestor and the post-K-Pg radiation of placentals. Science.

[9] Mitchell KJ, Pratt RC, Watson LN, Gibb GC, Llamas B, Kasper M, Edson J, Hopwood B, Male D, Armstrong KN, Meyer M, Hofreiter M, Austin J, Donnellan SC, Lee MS, Phillips MJ, Cooper A (2014). Molecular phylogeny, biogeography, and habitat preference evolution of Marsupials. Mol Biol Evol.

[10] Foley NM, Springer MS, Teeling EC (2016). Mammal madness: is the mammal tree of life not yet resolved?. Philos Trans R Sci.

[11] Tarver JE, Dos Reis M, Mirarab S, Moran RJ, Parker S, O’Reilly JE, King BL, O’Connell MJ, Asher RJ, Warnow T, Peterson KJ, Donoghue PC, Pisani D (2016). The interrelationships of placental mammals and the limits of phylogenetic inference. Genome Biol Evol.

[12] Marcus E, Hingst-Zaher E, Zaher H (2000). Application of landmark morphometrics to skulls representing the orders of living mammals. Hystrix.

[13] Brugmann SA, Kim J, Helms JA (2006). Looking different: understanding diversity in facial form. Am J Med Genet A..

[14] Goswami A (2006). Cranial modularity shifts during mammalian evolution. Am Nat.

[15] Porto A, de Oliveira FB, Shirai LT, De Conto V, Marroig G (2009). The Evolution of modularity in the mammalian skull I: morphological integration patterns and magnitudes. Evol Biol.

[16] Koyabu D, Maier W, Sánchez-Villagra MR (2012). Paleontological and developmental evidence resolve the homology and dual embryonic origin of a mammalian skull bone, the interparietal. Proc Natl Acad Sci USA.

[17] Cardini A, Polly PD (2013). Larger mammals have longer faces because of size-related constraints on skull form. Nat Commun.

[18] Koyabu D, Werneburg I, Morimoto N, Zollikofer CP, Forasiepi AM, Endo H, Kimura J, Ohdachi SD, Truong Son N, Sánchez-Villagra MR (2014). Mammalian skull heterochrony reveals modular evolution and a link between cranial development and brain size. Nat Commun.

[19] Jiang R, Bush JO, Lidral AC (2006). Development of the upper lip: morphogenetic and molecular mechanisms. Dev Dyn.

[20] Tehiler K (1989). The house mouse: atlas of embryonic development.

[21] O’Rahilly R, Müller F (2007). The development of the neural crest in the human. J Anat.

[22] Reid BS, Yang H, Melvin VS, Taketo MM, Williams T (2011). Ectodermal Wnt/β-catenin signaling shapes the mouse face. Dev Biol.

[23] Medio M, Yeh E, Popelut A, Babajko S, Berdal A, Helms JA (2012). Wnt/β-catenin signaling and Msx1 promote outgrowth of the maxillary prominences. Front Physiol.

[24] Lipinski RJ, Holloway HT, O’Leary-Moore SK, Ament JJ, Pecevich SJ, Cofer GP, Budin F, Everson JL, Johnson GA, Sulik KK (2014). Characterization of subtle brain abnormalities in a mouse model of hedgehog pathway antagonist-induced cleft lip and palate. PLoS ONE.

[25] Kaucka M, Ivashkin E, Gyllborg D, Zikmund T, Tesarova M, Kaiser J, Xie M, Petersen J, Pachni V, Nicolis SK, Yu T, Sharpe P, Arenas E, Brismar H, Blom H, Clevers H, Suter U, Chagin AS, Fried K, Hellander A, Adameyko I (2016). Analysis of neural crest-derived clones reveals novel aspects of facial development. Sci Adv.

[26] Juriloff DM, Harris MJ (2008). Mouse genetic models of cleft lip with or without cleft palate. Birth Defects Res A Clin Mol Teratol.

[27] Jugessur A, Farlie P, Kilpatrick N (2009). The genetics of isolated orofacial clefts: From genotypes to subphenotypes. Oral Dis.

[28] Dixon MJ, Marazita ML, Beaty TH, Murray JC (2011). Cleft lip and palate: Synthesizing genetic and environmental influences. Nat Rev Genet.

[29] Lan Y, Ryan RC, Zhang Z, Bullard AA, Bush JO, Maltby KM, Lidral AC, Jiang R (2006). Expression of *Wnt9b* and activation of canonical Wnt signaling during midfacial morphogenesis in mice. Dev Dyn.

[30] Bonilla-Claudio M, Wang J, Bai Y, Klysik E, Selever J, Martin JF (2012). Bmp signaling regulates a dose-dependent transcriptional program to control facial skeletal development. Development.

[31] Bakker ERM, Raghoebir L, Franken PF, Helvensteijn W, van Gurp L, Meijlink F, van der Valk MA, Rottier RJ, Kuipers EJ, van Veelen W, Smits R (2012). Induced Wnt5a expression perturbs embryonic outgrowth and intestinal elongation, but is well-tolerated in adult mice. Dev Biol.

[32] Ho HY, Susman MW, Bikoff JB, Ryu YK, Jonas AM, Hu L, Kuruvilla R, Greenberg ME (2012). Wnt5a-Ror-Dishevelled signaling constitutes a core developmental pathway that controls tissue morphogenesis. Proc Natl Acad Sci USA.

[33] Amerongen RV, Fuerer C, Mizutani M, Nusse R (2012). Wnt5a can both activate and repress Wnt/β-catenin signaling during mouse embryonic development. Dev Biol.

[34] Bush JO, Jiang R (2012). Palatogenesis: morphogenetic and molecular mechanisms of secondary palate development. Development.

[35] Noda K, Nakamura T, Komatsu Y (2015). Fibulin-5 deficiency causes developmental defect of premaxillary bone in mice. Biochem Biophys Res Commun.

[36] Yanagisawa H, Schluterman MK, Breken RA (2009). Fibulin-5, an integrin-binding matricellular protein: its function in development and disease. J Cell Commun Signal.

[37] Lan Y, Ovitt CE, Cho E, Maltby KM, Wang Q, Jiang R (2004). Odd-skipped related 2 (*Osr2*) encodes a key intrinsic regulator of secondary palate growth and morphogenesis. Development.

[38] Baek JA, Lan Y, Liu H, Maltby KM, Mishina Y, Jiang R (2011). Bmpr1a signaling plays critical roles in palatal shelf growth and palatal bone formation. Dev Biol.

[39] Pauws E, Hoshino A, Bentley L, Prajapati S, Keller C, Hammond P, Martinez-Barbera J, Moore GE, Stanier P (2009). *Tbx22*^*null*^ mice have a submucous cleft palate due to reduced palatal bone formation and also display ankyloglossia and choanal atresia phenotypes. Hum Mol Genet.

[40] Abzhanov A, Kuo WP, Hartman C, Grant BR, Grant PR, Tabin CJ (2006). The calmodulin pathway and evolution of elongated beak morphology in Darwin’s finches. Nature.

[41] Parsons KJ, Albertson RC (2009). Roles for Bmp4 and Cam1 in shaping the jaw: Evo-Devo and beyond. Annu Rev Genet.

[42] Knief U, Schielzeth H, Kempenaers B, Ellegren H, Forstmeier W (2012). QTL and quantitative genetic analysis of beak morphology reveals patterns of standing genetic variation in an Estrildid finch. Mol Ecol.

[43] Gunter H, Koppermann C, Meyer A (2014). Revisiting de Beer’s textbook example of heterochrony and jaw elongation in fish: calmodulin expression reflects heterochronic growth, and underlies morphological innovation in the jaws of belonoid fishes. EvoDevo.

[44] Suda T, Kobayashi K, Jimi E, Udagawa N, Takahashi N (2001). The molecular basis of osteoblast differentiation and activation. Novartis Found Symp.

[45] Komori T (2005). Regulation of skeletal development by the Runx family of transcription factors. J Cell Biochem.

[46] Schroeder TM, Jensen ED, Westendorf JJ (2005). Runx2: A master organizer of gene transcription in developing and maturing osteoblasts. Birth Defects Res C Embryo Today.

[47] Tokita M, Chaeychomsri W, Siruntawineti J (2013). Skeletal gene expression in the temporal region of the reptilian embryos: implications for the evolution of reptilian skull morphology. Springerplus.

[48] Komori T (2002). Runx2, A multifunctional transcription factor in skeletal development. J Cell Biochem.

[49] Sears KE, Goswami A, Flynn JJ, Niswander LA (2007). The correlated evolution of Runx2 tandem repeats, transcriptional activity, and facial length in Carnivora. Evol Dev.

[50] Ziros PG, Basdra EK, Papavassiliou AG (2008). Runx2: of bone and stretch. Int J Biochem Cell Biol.

[51] Newton AH, Feigin CY, Pask AJ (2017). RUNX2 repeat variation does not drive craniofacial diversity in marsupials. BMC Evol Biol.

[52] Fondon JW, Garner HR (2004). Molecular origins of rapid and continuous morphological evolution. Proc Natl Acad Sci USA.

[53] Fondon JW, Garner HR (2007). Detection of length-dependent effects of tandem repeat alleles by 3-D geometric decomposition of craniofacial variation. Dev Genes Evol.

[54] Ritzman TB, Banovich N, Buss KP, Guida J, Rubel MA, Pinney J, Khang B, Ravosa MJ, Stone AC (2017). Facing the facts: the Runx2 gene is associated with variation in facial morphology in primates. J Hum Evol.

[55] Pointer MA, Kamilar JM, Warmuth V, Chester SGB, Delsuc F, Mundy NI, AsherEmail RJ, Bradley BJ (2012). RUNX2 tandem repeats and the evolution of facial length in placental mammals. BMC Evol Biol.

[56] Schoenebeck JJ, Hutchinson SA, Byers A, Beale HC, Carrington B, Faden DL, Rimbault M, Decker B, Kidd JM, Sood R, Boyko AR, Fondon JW, Wayne RK, Bustamante CD, Ciruna B, Ostrander EA (2012). Variation of BMP3 contributes to dog breed skull diversity. PLoS Genet.

[57] Abzhanov A, Protas M, Grant BR, Grant PR, Tabin C (2004). Bmp4 and morphological variation of beaks in Darwin’s Finches. Science.

[58] Campàs O, Mallarino R, Herrel A, Abzhanov A (2010). Brenner MP. Scaling and shear transformations capture beak shape variation in Darwin’s finches. Proc Natl Acad Sci USA.

[59] Mallarino R, Grant PR, Grant BR, Herrel A, Kuo WP, Abzhanova A (2011). Two developmental modules establish 3D beak-shape variation in Darwin’s finches. Proc Natl Acad Sci USA.

[60] Mallarino R, Campàs O, Fritz J, Burns K, Weeks OG, Brenner MP, Abzhanov A (2012). Closely related bird species demonstrate flexibility between beak morphology and underlying developmental programs. Proc Natl Acad Sci USA.

[61] Fritz JA, Brancale J, Tokita M, Burns KJ, Hawkins MB, Abzhanov A, Brenner MP (2014). Shared developmental programme strongly constrains beak shape diversity in songbirds. Nat Commun.

[62] Tokita M, Yano W, James HF, Abzhanov A (2017). Cranial shape evolution in adaptive radiations of birds: comparative morphometrics of Darwin’s finches and Hawaiian honeycreepers. Philos Trans R Soc Lond B Biol Sci.

[63] Fenton MB, Simmons NB (2014). Bats a world of science and mystery.

[64] Eick GN, Jacobs DS, Matthee CA (2005). A nuclear DNA phylogenetic perspective on the evolution of echolocation and historical biogeography of extant bats (Chiroptera). Mol Biol Evol.

[65] Teeling EC, Springer MS, Madsen O, Bates P, O’brien SJ, Murphy WJ (2005). A molecular phylogeny for bats illuminates biogeography and the fossil record. Science.

[66] Tsagkogeorga G, Parker J, Stupka E, Cotton JA, Rossiter SJ (2013). Phylogenomic analyses elucidate the evolutionary relationships of bats. Curr Biol.

[67] Shi JJ, Rabosky DL (2015). Speciation dynamics during the global radiation of extant bats. Evolution.

[68] Chen CH, Cretekos CJ, Rasweiler JJ, Behringer RR (2005). Hoxd13 expression in the developing limbs of the short-tailed fruit bat, *Carollia perspicillata*. Evol Dev.

[69] Sears KE, Behringer RR, Rasweiler JJ, Niswander LA (2006). Development of bat flight: morphologic and molecular evolution of bat wing digits. Proc Natl Acad Sci USA.

[70] Weatherbee SD, Behringer RR, Rasweiler JJ, Niswander LA (2006). Interdigital webbing retention in bat wings illustrates genetic changes underlying amniote limb diversification. Proc Natl Acad Sci.

[71] Cretekos CJ, Wang Y, Green ED, Martin JF, Rasweiler JJ, Behringer RR (2008). Regulatory divergence modifies limb length between mammals. Genes Dev.

[72] Hockman D, Cretekos CJ, Mason MK, Behringer RR, Jacobs DS, Illing N (2008). A second wave of Sonic hedgehog expression during the development of the bat limb. Proc Natl Acad Sci USA.

[73] Wang Z, Dong D, Ru B, Young RL, Han N, Guo T, Zhang S (2010). Digital gene expression tag profiling of bat digits provides robust candidates contributing to wing formation. BMC Genom.

[74] Tokita M, Abe T, Suzuki K (2012). The developmental basis of bat wing muscle. Nat Commun.

[75] Dai M, Wang Y, Fang L, Irwin DM, Zhu T, Zhang J, Zhang S, Wang Z (2014). Differential expression of Meis2, Mab21l2 and Tbx3 during limb development associated with diversification of limb morphology in mammals. PLoS ONE.

[76] Wang Z, Dai M, Wang Y, Cooper KL, Zhu T, Dong D, Zhang J, Zhang S (2014). Unique expression patterns of multiple key genes associated with the evolution of mammalian flight. Proc Biol Sci.

[77] Mason MK, Hockman D, Curry L, Cunningham TJ, Duester G, Logan M, Jacobs DS, Illing N (2015). Retinoic acid-independent expression of Meis2 during autopod patterning in the developing bat and mouse limb. Evodevo.

[78] Tokita M (2015). How the pterosaur got its wing. Biol Rev.

[79] Booker BM, Friedrich T, Mason MK, VanderMeer JE, Zhao J, Eckalbar WL, Logan M, Illing N, Pollard KS, Ahituv N (2016). Bat accelerated regions identify a bat forelimb specific enhancer in the HoxD locus. PLoS Genet.

[80] Eckalbar WL, Schlebusch SA, Mason MK, Gill Z, Parker AV, Booker BM, Nishizaki S, Muswamba-Nday C, Terhune E, Nevonen KA, Makki N, Friedrich T, VanderMeer JE, Pollard KS, Carbone L, Wall JD, Illing N, Ahituv N (2016). Transcriptomic and epigenomic characterization of the developing bat wing. Nat Genet.

[81] Haeckel E. Kunstformen der Natur. 1904.

[82] Dumont ER, Dávalos LM, Goldberg A, Santana SE, Rex K, Voigt CC (2011). Morphological innovation, diversification and invasion of a new adaptive zone. Proc Biol Sci.

[83] Baker RJ, Bininda-Emonds ORP, Mantilla-Meluk H, Porter CA, Van Den Bussche RA. Molecular timescale of diversification of feeding strategy and morphology in New World Leaf-Nosed Bats (Phyllostomidae): A phylogenetic perspective. In Evolutionary history of bats: fossils, molecules and morphology. Edited by Gunnell GF. Simmons NB. Cambridge: Cambridge University Press. 2012;385–409.

[84] Dumont ER (1999). The effect of food hardness on feeding behavior in frugivorous bats (Phyllostomidae): an experimental study. J Zool Lond.

[85] Dumont ER (2004). Patterns of diversity in cranial shape among plant-visiting bats. Acta Chiropterol.

[86] Santana SE, Grosse IR, Dumont ER (2012). Dietary hardness, loading behavior, and the evolution of skull form in bats. Evolution.

[87] Giannini NP, Simmons NB (2007). The Chiropteran Premaxilla: a reanalysis of morphological variation and its phylogenetic interpretation. Am Museum Novit.

[88] Orr DJA, Teeling EC, Puechmaille SJ, Finarelli JA (2016). Patterns of orofacial clefting in the facial morphology of bats: a possible naturally occurring model of cleft palate. J Anat.

[89] Phillips CD, Butler B, Fondon JW, Mantilla-Meluk H, Baker RJ (2013). Contrasting evolutionary dynamics of the developmental regulator PAX9, among bats, with evidence for a novel post-transcriptional regulatory mechanism. PLoS ONE.

[90] Shapiro MD, Bell MA, Kingsley DM (2006). Parallel genetic origins of pelvic reduction in vertebrates. Proc Natl Acad Sci USA.

[91] Kohlsdorf T, Cummings MP, Lynch VJ, Stopper GF, Takahashi K, Wagner GP (2008). A molecular footprint of limb loss: sequence variation of the autopodial identity gene Hoxa-13. J Mol Evol.

[92] Woltering JM, Vonk FJ, Müller H, Bardine N, Tuduce IL, de Bakker MA, Knöchel W, Sirbu IO, Durston AJ, Richardson MK (2009). Axial patterning in snakes and caecilians: evidence for an alternative interpretation of the Hox code. Dev Biol.

[93] Mallarino R, Henegar C, Mirasierra M, Manceau M, Schradin C, Vallejo M, Beronja S, Barsh GS, Hoekstra HE (2016). Developmental mechanisms of stripe patterns in rodents. Nature.

[94] Sears KE (2014). Differences in growth generate the diverse palate shapes of new world leaf-nosed Bats (Order Chiroptera, Family Phyllostomidae). Evol Biol.

[95] Cretekos CJ, Weatherbee SD, Chen CH, Badwaik NK, Niswander L, Behringer RR, Rasweiler JJ (2005). Embryonic staging system for the short-tailed fruit bat, *Carollia perspicillata*, a model organism for the mammalian order Chiroptera, based upon timed pregnancies in captive-bred animals. Dev Dyn.

[96] Giannini N, Goswami A, Sánchez-Villagra MR (2006). Development of integumentary structures in *Rousettus amplexicaudatus* (Mammalia: Chiroptera: Pteropodidae) during late-embryonic and fetal stages. J Mammal.

[97] Tokita M (2006). Normal embryonic development of the *Japanese pipistrelle*, *Pipistrellus abramus*. Zoology..

[98] Hockman D, Mason MK, Jacobs DS, Illing N (2009). The role of early development in mammalian limb diversification: a descriptive comparison of early limb development between the natal long-fingered bat (*Miniopterus natalensis*) and the mouse (*Mus musculus*). Dev Dyn.

[99] Wang Z, Han N, Racey PA, Ru B, He G (2010). A comparative study of prenatal development in *Miniopterus schreibersii fuliginosus*, *Hipposideros armiger* and *H*. *pratti*. BMC Dev Biol.

[100] Paksuz EP, Hayretdag S, Olgun K (2017). Prenatal development in greater mouse- eared bat, *Myotis myotis* (Borkhausen, 1797) (Chiroptera, Vespertilionidae). Anat Histol Embryol.

[101] Chen MY, Liang D, Zhang P (2017). Phylogenomic Resolution of the Phylogeny of Laurasiatherian Mammals: Exploring Phylogenetic Signals within Coding and Noncoding Sequences. Genome Biol Evol..

[102] De Moraes DA, Hingst-Zaher E, Maarcus L, Cerqueria R (2000). A geometric morphometric analysis of cranial and mandibular shape variation of didelphid marsupials. Hyatrix It J Mamm..

[103] Goswami A, Polly PD, Mock OB, Sánchez-Villagra MR (2012). Shape, variance and integration during craniogenesis: contrasting marsupial and placental mammals. J Evol Biol.

[104] Prevosti FJ, Turazzini GF, Ercoli MD, Hingst-Zaher E (2012). Mandible shape in marsupial and placental carnivorous mammals: a morphological comparative study using geometric morphometrics. Zool J Linn Soc..

[105] Bennett CV, Goswami A (2013). Statistical support for the hypothesis of developmental constraint in marsupial skull evolution. BMC Biol.

[106] De Iuliis G, Bargo MS. Vizcaíno SF. Variation in skull morphology and mastication in the fossil giant armadillos *Pampatherium* spp. And allied genera (Mammalia: Xenarthra: Pampatheriidae), with comments on their systematics and distribution. J Verterbr Paleontol. 2000;20:743-54.

[107] Hautier L, Billet G, Eastwood B, Lane J (2014). Patterns of morphological variation of extant sloth skulls and their implication for future conservation efforts. Anat Rec.

[108] Hossotani CMS, Ragusa-Netto J, e Luna HS. Skull morphometry and vault sutures of *Myrmecophaga tridactyla* and *Tamandua tetradactyla*. Iheringia, Série Zoologia. 2017;107:e2017038.

[109] Panchetti F, Scalici M, Carpaneto GM, Gibertini G (2008). Shape and size variations in the cranium of elephant-shrews: a morphometric contribution to a phylogenetic debate. Zoomorphology.

[110] Scalici M, Panchetti F (2011). Morphological cranial diversity contributes to phylogeny in soft-furred sengis (Afrotheria, Macroscelidea). Zoology..

[111] Finlay S, Cooper N (2015). Morphological diversity in tenrecs (Afrosoricida, Tenrecidae): comparing tenrec skull diversity to their closest relatives. PeerJ..

[112] Paunović G, Bogićević K, Urošević A. Mandible shape differentiation between *Mammuthus trogontherii* and *M.* *primigenius* and mandible shape ontogeny in *M.* *primigenius* specimens from Serbia: A preliminary explorative geometric morphometric study. Quant Int. 2017;443:212-20.

[113] Barros H, Meirelles ACO, Luna FO, Marmontel M, Cordeiro-Esterla P, Santos N, Astúa D (2017). Cranial and chromosomal geographic variation in manatees (Mammalia: Sirenia: Trichechidae) with the description of the Antillean manatee karyotype in Brazil. J Zool Syst Ecol Res..

[114] Rohlf FJ, Loy A, Corti M (1996). Morphometric analysis of old world Talpidae (Mammalia, Insectivora) using Partial-warp scores. Syst Biol.

[115] Loy A, Capula M, Palombi A, Capanna E (2001). Genetic and morphometric evidence of introgression between two species of moles (Insectivora: *Talpa europaea* and *Talpa romana*) in central Italy. J Zool Lond..

[116] Barrow E, Macleod N (2008). Shape variation in the mole dentary (Talpidae: Mammalia). Zool J Linn Soc..

[117] Ventura J, López-Fuster MJ (2010). Geometric morphometrics of the mandible in the Iberian desman, *Galemys pyrenaicus* (Mammalia: Soricomorpha): Is there a significant variation in form during post-weaning life?. Mamm Biol..

[118] Shchipanov NA, Voyta LL, Bobretsov AV, Kuprianova IF (2014). Intra-species structuring in the common shrew *Sorex araneus* (Lipotyphla: Soricidae) in European Russia: morphometric variability could give evidence of limitation of interpopulation migration. Russian J Theriol..

[119] Shchipanova NA, Sychevaa VB, Tumasyan FA (2016). Morphometric distances and population structuring in the common shrew *Sorex araneus* L. (Lipotyphla: Soricidae). Biology Bulletin..

[120] Bornholdt R, Oliveira LR, Fabián ME (2008). Size and shape variability in the skull of Myotis nigricans (Schinz, 1821) (Chiroptera: Vespertilionidae) from two geographic areas in Brazil. Braz J Biol..

[121] Sorensen DW, Butkus C, Cooper LN, Cretekos CJ, Rasweiler JJ, Sears KE (2014). Palate variation and evolution in New World leaf-nosed and Old World fruit bats (Order Chiroptera). Evol Biol.

[122] Zuccarelli MD (2004). Comparative morphometric analysis of captive vs. wild African lion (*Panthera leo*) skulls. Bios..

[123] Slater GJ, Van Valkenburgh B (2009). Allometry and performance: the evolution of skull form and function in felids. J Evol Biol.

[124] Schutz H, Polly PD, Krieger JD, Guralnick RP (2009). Differential sexual dimorphism: size and shape in the cranium and pelvis of grey foxes (*Urocyon*). Biol J Linn Soc.

[125] Goswami A, Polly PD (2010). The influence of modularity on cranial morphological disparity in Carnivora and Primates (Mammalia). PLoS ONE.

[126] Drake AG, Klingenberg CP (2010). Large-scale diversification of skull shape in domestic dogs: disparity and modularity. Am Nat.

[127] Figueirido B1, Serrano-Alarcón FJ, Slater GJ, Palmqvist P. Shape at the cross-roads: homoplasy and history in the evolution of the carnivoran skull towards herbivory. J Evol Biol. 2010;23:2579-94. 10.1111/j.1420-9101.2010.02117.x 20942824

[128] Milenković M, Šipetić VJ, Blagojević J, Tatović S, Vujošević M (2010). Skull variation in Dinaric-Balkan and Carpathian gray wolf populations revealed by geometric morphometric approaches. J Mammal.

[129] Tanner JB, Zelditch ML, Lundrigan BL, Holekamp KE (2010). Ontogenetic change in skull morphology and mechanical advantage in the spotted hyena (*Crocuta crocuta*). J Morphol.

[130] Goswami A, Milne N, Wroe S (2011). Biting through constraints: cranial morphology, disparity and convergence across living and fossil carnivorous mammals. Proc Biol Sci..

[131] Drake AG (2011). Dispelling dog dogma: an investigation of heterochrony in dogs using 3D geometric morphometric analysis of skull shape. Evol Dev..

[132] Figueirido B, Palmqvist P, Pérez-Claros JA, Dong W (2011). Cranial shape transformation in the evolution of the giant panda (*Ailuropoda melanoleuca*). Naturwissenschaften.

[133] Asahara M (2013). Shape variation in the skull and lower carnassial in a wild population of raccoon dog (Nyctereutes procyonoides). Zoolog Sci.

[134] Figueirido B, Tseng ZJ, Martín-Serra A (2013). Skull shape evolution in durophagous carnivorans. Evolution.

[135] Schoenebeck JJ, Ostrander EA (2013). The genetics of canine skull shape variation. Genetics.

[136] Piras P, Maiorino L, Teresi L, Meloro C, Lucci F, Kotsakis T, Raia P (2013). Bite of the cats: relationships between functional integration and mechanical performance as revealed by mandible geometry. Syst Biol.

[137] Asahara M (2014). Shape variation in the skull within and between wild populations of the raccoon dog (*Nyctereutes procyonoides*) in Japan. Mammal Study..

[138] Curtis AA, Van Valkenburgh B (2014). Beyond the sniffer: frontal sinuses in Carnivora. Anat Rec (Hoboken).

[139] Meachen JA, Janowicz AC, Avery JE, Sadleir RW (2014). Ecological changes in Coyotes (Canis latrans) in response to the ice age megafaunal extinctions. PLoS ONE.

[140] Jones KE, Smaers JB, Goswami A (2015). Impact of the terrestrial-aquatic transition on disparity and rates of evolution in the carnivoran skull. BMC Evol Biol.

[141] Tseng ZJ, Flynn JJ (2015). Are cranial biomechanical simulation data linked to known diets in extant taxa? A method for applying diet-biomechanics linkage models to infer feeding capability of extinct species. PLoS ONE.

[142] Lau AC, Asahara M, Han SY, Kimura J (2016). Sexual dimorphism of the Eurasian otter (Lutra lutra) in South Korea: Craniodental geometric morphometry. J Vet Med Sci.

[143] de Moura Bubadué J, Cáceres N, Dos Santos Carvalho R, Meloro C (2016). Ecogeographical Variation in Skull Shape of South-American Canids: Abiotic or Biotic Processes?. Evol Biol.

[144] Bertolini F, Gandolfi B, Kim ES, Haase B, Lyons LA, Rothschild MF (2016). Evidence of selection signatures that shape the Persian cat breed. Mamm Genome.

[145] Lau AC, Asahara M, Han SY, Kimura J (2017). Geographic variation of craniodental morphology of the Eurasian otter (*Lutra lutra*) in East Asia. J Vet Med Sci.

[146] Geiger M, Evin A, Sánchez-Villagra MR, Gascho D, Mainini C, Zollikofer CPE (2017). Neomorphosis and heterochrony of skull shape in dog domestication. Sci Rep..

[147] Yoshida H, Shirakihara K, Shirakihara M, Takemura A (1995). Geographic variation in the skull morphology of the finless porpoise *Neophocaena phocaenoides* in Japan waters. Fish Sci.

[148] Monteiro-Filho ELA, Monteiro LR, Reis SF (2002). Skull shape and size divergence in dolphins of the genus Sotalia: a tridimensional morphometric analysis. J Mammal.

[149] Amaral AR, Coelho MM, Marugán-Lobón J, Rohlf FJ (2009). Cranial shape differentiation in three closely related delphinid cetacean species: Insights into evolutionary history. Zoology..

[150] Gutstein CS, Cozzuol MA, Vargas AO, Suárez MA, Schultz CL, Rubilar-Rogers DR (2009). Patterns of skull variation of *Brachydelphis* (Cetacea, Odontoceti) from the Neogene of the southeastern pacific. J Mammal.

[151] Galatius A, Berta A, Frandsen MS, Goodall RNP (2011). Interspecific variation of ontogeny and skull shape among porpoises (Phocoenidae). J Morphol.

[152] Loy A, Tamburelli A, Carlini R, Slice DE (2011). Craniometric variation of some Mediterranean and Atlantic populations of *Stenella coeruleoalba* (Mammalia, Delphinidae): A three-dimensional geometric morphometric analysis. Marine Mammal Science..

[153] Parés-Casanova PM (2013). Fabre – Anatomia L. Size and shape variability in the skull of the bottlenose dolphin, *Tursiops truncatus* (Montagu, 1821). Anat Histol Embryol.

[154] Del Castillo DL, Flores DA, Cappozzo HL (2014). Ontogenetic development and sexual dimorphism of franciscana dolphin skull: A 3D geometric morphometric approach. J Morphol.

[155] Owen J, Dobney K, Evin A, Cucchi T, Larson G, Vidarsdottir US (2014). The zooarchaelogical application of quantifying cranial shape differences in wild boar and domestic pig (*Sus scrofa*) using 3D geometric morphometrics. J Archaeol Sci.

[156] Gol’din P, Vishnyakova K. Habitat shapes skull profile of small cetaceans: evidence from geographical variation in Black Sea harbour porpoises (*Phocoena phocoena relicta*). Zoomorphology. 2016;135:387-93.

[157] Evin A, Owen J, Larson G, Debiais-Thibaud M, Cucchi T, Vidarsdottir US, Dobney K (2017). A test for paedomorphism in domestic pig cranial morphology. Biol Lett.

[158] Endo H, Rashdi ABM, Yamagiwa D, Yamada J (2000). Principal component analysis of the common tree shrew skulls from Laos, Thailand and Malaysia. National Science Museum monographs..

[159] Yusoff AM, Kumaran JV, Mohd Tahir NFD, Mokhtar SI. Preliminary study of skull polymorphims of tupaia glis in Peninsular Malaysia by using morphoj. Jurnal Teknologi (Sciences & Engineering). 2015;72:67-9.

[160] Sargis EJ, Woodman N, Morningstar NC, Bell TN, Olson LE (2017). Skeletal variation and taxonomic boundaries among mainland and island populations of the common treeshrew (Mammalia: Scandentia: Tupaiidae). Biol J Linn Soc.

[161] Corti M, Di Giuliomaria C, Verheyen W. Three-dimensional geometric morphometrics of the African genus *Lophuromys* (Rodenta Muridae). Hystrix It J Mamm. 2000;11.

[162] Corti M, Aguilera M, Capanna E. Size and shape changes in the skull accompanying speciation of South American spiny rats (Rodentia: *Proechimys spp*.). J Zool Lond. 2001;253:537-47.

[163] Klingenberg CP, Leamy LJ, Routman EJ, Cheverud JM (2001). Genetic architecture of mandible shape in mice: effects of quantitative trait loci analyzed by geometric morphometrics. Genetics.

[164] Dobigny G, Baylac M, Denys C (2002). Geometric morphometrics, neural networks and diagnosis of sibling *Taterillus* species (Rodentia, Gerbillinae). Biol J Linn Soc.

[165] Klingenberg CP, Mebus K, Auffray JC (2003). Developmental integration in a complex morphological structure: how distinct are the modules in the mouse mandible?. Evol Dev..

[166] Klingenberg CP1, Leamy LJ, Cheverud JM. Integration and modularity of quantitative trait locus effects on geometric shape in the mouse mandible. Genetics. 2004;166:1909-21. 10.1534/genetics.166.4.1909 PMC1470826 15126408

[167] Zelditch ML1, Lundrigan BL, Garland T Jr. Developmental regulation of skull morphology. I. Ontogenetic dynamics of variance. Evol Dev. 2004;6:194-206. 10.1111/j.1525-142X.2004.04025.x 15099307

[168] Kawakami M, Yamamura K (2008). Cranial bone morphometric study among mouse strains. BMC Evol Biol.

[169] Fornel R, Cordeiro-Estrela P, De Freitas TR (2010). Skull shape and size variation in *Ctenomys minutus* (Rodentia: Ctenomyidae) in geographical, chromosomal polymorphism, and environmental contexts. Biol J Linn Soc.

[170] Boell L, Tautz D (2011). Micro-evolutionary divergence patterns of mandible shapes in wild house mouse (*Mus musculus*) populations. BMC Evol Biol.

[171] Cox PG, Fagan MJ, Rayfield EJ, Jeffery N (2011). Finite element modelling of squirrel, guinea pig and rat skulls: using geometric morphometrics to assess sensitivity. J Anat.

[172] Jamniczky HA, Hallgrímsson B (2011). Modularity in the skull and cranial vasculature of laboratory mice: implications for the evolution of complex phenotypes. Evol Dev..

[173] Burgio G, Baylac M, Heyer E, Montagutelli X. Exploration of the genetic organization of morphological modularity on the mouse mandible using a set of interspecific recombinant congenic strains between C57BL /6 and mice of the *Mus spretus* species. Genes Genomes Genetics. 2012;2-1257-68. 10.1534/g3.112.003285 PMC3464118 23050236

[174] Hautier L, Lebrun R, Cox PG (2012). Patterns of covariation in the masticatory apparatus of hystricognathous rodents: implications for evolution and diversification. J Morphol.

[175] Paradis MR, Raj MT, Boughner JC (2013). Jaw growth in the absence of teeth: the developmental morphology of edentulous mandibles using the p63 mouse mutant. Evol Dev..

[176] Gonzalez PN, Kristensen E, Morck DW, Boyd S, Hallgrímsson B (2013). Effects of growth hormone on the ontogenetic allometry of craniofacial bones. Evol Dev..

[177] Christians JK, de Zwaan DR, Fung SH (2013). Pregnancy associated plasma protein A2 (PAPP-A2) affects bone size and shape and contributes to natural variation in postnatal growth in mice. PLoS ONE.

[178] Casanovas-Vilar I, van Dam J (2013). Conservatism and adaptability during squirrel radiation: what is mandible shape telling us?. PLoS ONE.

[179] Stolz JF, Gonçalves GL, Leipnitz L, Freitas TR (2013). DNA-based and geometric morphometric analysis to validate species designation: a case study of the subterranean rodent *Ctenomys bicolor*. Genet Mol Res.

[180] Anderson PS, Renaud S, Rayfield EJ (2014). Adaptive plasticity in the mouse mandible. BMC Evol Biol.

[181] Parsons TE, Weinberg SM, Khaksarfard K, Howie RN, Elsalanty M, Yu JC, Cray JJ (2014). Craniofacial shape variation in *Twist1*^+*/*−^ mutant mice. Anat Rec (Hoboken).

[182] Pallares LF, Harr B, Turner LM, Tautz D (2014). Use of a natural hybrid zone for genomewide association mapping of craniofacial traits in the house mouse. Mol Ecol.

[183] Lu X, Ge D, Xia L, Huang C, Yang Q (2014). Geometric morphometric study of the skull shape diversification in Sciuridae (Mammalia, Rodentia). Integr Zool..

[184] Singh N, Dutka T, Devenney BM, Kawasaki K, Reeves RH, Richtsmeier JT (2015). Acute upregulation of hedgehog signaling in mice causes differential effects on cranial morphology. Dis Model Mech..

[185] Lalis A, Evin A, Janier M, Koivogui L, Denys C (2015). Host evolution in Mastomys natalensis (Rodentia: Muridae): An integrative approach using geometric morphometrics and genetics. Integr Zool..

[186] Walsh RE, Aprígio Assis AP, Patton JL, Marroig G, Dawson TE, Lacey EA (2016). Morphological and dietary responses of chipmunks to a century of climate change. Glob Chang Biol..

[187] Pallares LF, Turner LM, Tautz D (2016). Craniofacial shape transition across the house mouse hybrid zone: implications for the genetic architecture and evolution of between-species differences. Dev Genes Evol.

[188] Quintela FM, Fornel R, Freitas TR (2016). Geographic variation in skull shape of the water rat *Scapteromys tumidus* (Cricetidae, Sigmodontinae): isolation-by-distance plus environmental and geographic barrier effects?. An Acad Bras Cienc..

[189] Percival CJ, Liberton DK, Pardo-Manuel de Villena F, Spritz R, Marcucio R, Hallgrímsson B (2016). Genetics of murine craniofacial morphology: diallel analysis of the eight founders of the Collaborative Cross. J Anat.

[190] Vasil’ev AG, Bolshakov VN, Evdokimov NG, Sineva NV. Morphological diversity of mole vole mono- and polymorphic populations: Does Chernov’s “compensation principle” work within a population? Dokl Biol Sci. 2016;468:118-21. 10.1134/S0012496616030030 27411822

[191] Pavličev M, Mitteroecker P, Gonzalez PM, Rolian C, Jamniczky H, Villena FP, Marcucio R, Spritz R, Hallgrimsson B (2016). Development Shapes a Consistent Inbreeding Effect in Mouse Crania of Different Line Crosses. J Exp Zool B Mol Dev Evol..

[192] Singh N, Albert FW, Plyusnina I, Trut L, Pӓӓbo S, Harvati K (2017). Facial shape differences between rats selected for tame and aggressive behaviors. PLoS ONE.

[193] Abramov SA, Lopatina NV, Litvinov YN (2017). Cranial size and shape variation in isolated populations of the Olkhon mountain vole (Alticola olchonensis Litvinov, 1960). Zoology (Jena)..

[194] Caumul R, Polly PD (2005). Phylogenetic and environmental components of morphological variation: skull, mandible, and molar shape in marmots (*Marmota*, Rodentia). Evolution.

[195] Ge D, Lv X, Xia L, Huang C, Yang Q (2012). Geometric morphometric of postnatal size and shape changes in the cranium of cape hare (Lagomorpha, Leporidea, *Lepus capensis*). Acta Thariologica Sinica..

[196] Kraatz BP, Sherratt E, Bumacod N, Wedel MJ (2015). Ecological correlates to cranial morphology in Leporids (Mammalia, Lagomorpha). PeerJ..

[197] Ge D, Yao L, Xia L, Zhang Z, Yang Q (2015). Geometric morphometric analysis of skull morphology reveals loss of phylogenetic signal at the generic level in extant lagomorphs (Mammalia: Lagomorpha). Contributions to Zoology..

[198] Kraatz B, Sherratt E (2016). Evolutionary morphology of the rabbit skull. PeerJ..

[199] De León MSP, Zollikofer CPE (2001). Neanderthal cranial ontogeny and its implications for late hominid diversity. Nature.

[200] Singleton M (2002). Patterns of cranial shape variation in the Papionini (Primates: Cercopithecinae). J Hum Evol.

[201] Hallgrímsson B, Willmore K, Dorval C, Cooper DM (2004). Craniofacial variability and modularity in macaques and mice. J Exp Zool B Mol Dev Evol..

[202] Perez SI, Bernal V, Gonzalez PN (2006). Differences between sliding semi-landmark methods in geometric morphometrics, with an application to human craniofacial and dental variation. J Anat.

[203] Cardini A, Elton S (2008). Variation in guenon skulls (I): species divergence, ecological and genetic differences. J Hum Evol.

[204] Shirai LT, Marroig G (2010). Skull modularity in neotropical marsupials and monkeys: size variation and evolutionary constraint and flexibility. J Exp Zool B Mol Dev Evol..

[205] Bennett CV, Goswami A (2012). Morphometric analysis of cranial shape in fossil and recent euprimates. Anat Res Int..

[206] Hayashi K, Saitoh S, Mizoguchi I (2012). Morphological analysis of the skeletal remains of Japanese females from the Ikenohata-Shichikencho site. Eur J Orthod.

[207] Robinson C (2012). Geometric morphometric analysis of mandibular shape diversity in *Pan*. J Hum Evol.

[208] Neaux D, Guy F, Gilissen E, Coudyzer W, Vignaud P, Ducrocq S (2013). Facial orientation and facial shape in extant great apes: a geometric morphometric analysis of covariation. PLoS ONE.

[209] Ito T, Nishimura T, Takai M (2014). Ecogeographical and phylogenetic effects on craniofacial variation in macaques. Am J Phys Anthropol.

[210] Pérez-Claros JA, Jiménez-Arenas JM, Palmqvist P (2015). Neurocranium versus Face: A Morphometric Approach with Classical Anthropometric Variables for Characterizing Patterns of Cranial Integration in Extant Hominoids and Extinct Hominins. PLoS ONE.

[211] Neaux D, Gilissen E, Coudyzer W, Guy F (2015). Integration between the face and the mandible of *Pongo* and the evolution of the craniofacial morphology of orangutans. Am J Phys Anthropol.

[212] Fleagle JG, Gilbert CC, Baden AL (2016). Comparing primate crania: The importance of fossils. Am J Phys Anthropol.

[213] Martinez-Maza C, Freidline SE, Strauss A, Nieto-Diaz M (2016). Bone Growth Dynamics of the Facial Skeleton and Mandible in *Gorilla gorilla* and *Pan troglodytes*. Evol Biol.

[214] Schroeder L, Scott JE, Garvin HM, Laird MF, Dembo M, Radovčić D, Berger LR, de Ruiter DJ, Ackermann RR (2017). Skull diversity in the Homo lineage and the relative position of *Homo naledi*. J Hum Evol.

[215] Liu W, Sun X, Braut A, Mishina Y, Behringer RR, Mina M, Martin JF (2005). Distinct functions for Bmp signaling in lip and palate fusion in mice. Development..

[216] Metzis V, Courtney AD, Kerr MC, Ferguson C, Rondón Galeano MC, Parton RG, Wainwright BJ, Wicking C (2013). Patched1 is required in neural crest cells for the prevention of orofacial clefts. Hum Mol Genet.

[217] Jeong J, Mao J, Tenzen T, Kottmann AH, McMahon AP (2004). Hedgehog signaling in the neural crest cells regulates the patterning and growth of facial primordia. Genes Dev.

[218] Green RM, Feng W, Phang T, Fish JL, Li H, Spritz RA, Marcucio RS, Hooper J, Jamniczky H, Hallgrímsson B, Williams T (2015). *Tfap2a*-dependent changes in mouse facial morphology result in clefting that can be ameliorated by a reduction in Fgf8 gene dosage. Dis Model Mech..

[219] Niemann S, Zhao C, Pascu F, Stahl U, Aulepp U, Niswander L, Weber JL, Müller U (2004). Homozygous *WNT3* mutation causes tetra-amelia in a large consanguineous family. Am J Hum Genet.

[220] Juriloff DM, Harris MJ, McMahon AP, Carroll TJ, Lidral AC (2006). *Wnt9b* is the mutated gene involved in multifactorial nonsyndromic cleft lip with or without cleft palate in A/WySn mice, as confirmed by a genetic complementation test. Birth Defects Res A Clin Mol Teratol..

[221] Wu W, Gu S, Sun C, He W, Xie X, Li X, Ye W, Qin C, Chen Y, Xiao J, Liu C (2015). Altered FGF Signaling Pathways Impair Cell Proliferation and Elevation of Palate Shelves. PLoS ONE.

[222] Brugmann SA, Allen NC, James AW, Mekonnen Z, Madan E, Helms JA (2010). A primary cilia-dependent etiology for midline facial disorders. Hum Mol Genet.

[223] Satokata I, Maas R (1994). *Msx1* deficient mice exhibit cleft palate and abnormalities of craniofacial and tooth development. Nat Genet.

[224] Zhang Z, Song Y, Zhao X, Zhang X, Fermin C, Chen Y (2002). Rescue of cleft palate in *Msx1*-deficient mice by transgenic *Bmp4* reveals a network of BMP and Shh signaling in the regulation of mammalian palatogenesis. Development..

[225] Meester-Smoor MA, Vermeij M, van Helmond MJ, Molijn AC, van Wely KH, Hekman AC, Vermey-Keers C, Riegman PH, Zwarthoff EC (2005). Targeted disruption of the Mn1 oncogene results in severe defects in development of membranous bones of the cranial skeleton. Mol Cell Biol.

[226] Yu L, Gu S, Alappat S, Song Y, Yan M, Zhang X, Zhang G, Jiang Y, Zhang Z, Zhang Y, Chen Y (2005). *Shox2*-deficient mice exhibit a rare type of incomplete clefting of the secondary palate. Development..

[227] Ito Y, Yeo JY, Chytil A, Han J, Bringas P, Nakajima A, Shuler CF, Moses HL, Chai Y (2003). Conditional inactivation of *Tgfbr2* in cranial neural crest causes cleft palate and calvaria defects. Development..

[228] Lack JB, Van Den Bussche RA (2010). Identifying the confounding factors in resolving phylogenetic relationships in Vespertilionidae. J Mammal.

